# Tryptophan metabolites suppress the Wnt pathway and promote adverse limb events in chronic kidney disease

**DOI:** 10.1172/JCI142260

**Published:** 2022-01-04

**Authors:** Nkiruka V. Arinze, Wenqing Yin, Saran Lotfollahzadeh, Marc Arthur Napoleon, Sean Richards, Joshua A. Walker, Mostafa Belghasem, Jonathan D. Ravid, Mohamed Hassan Kamel, Stephen A. Whelan, Norman Lee, Jeffrey J. Siracuse, Stephan Anderson, Alik Farber, David Sherr, Jean Francis, Naomi M. Hamburg, Nader Rahimi, Vipul C. Chitalia

**Affiliations:** 1 Division of Vascular and Endovascular Surgery, Department of Surgery,; 2 Renal Section, Department of Medicine,; 3 Whitaker Cardiovascular Institute,; 4 Department of Pathology and Laboratory Medicine, and; 5 School of Medicine, Boston University School of Medicine (BUSM), Boston, Massachusetts, USA.; 6 Chemical Instrumentation Center, Department of Chemistry, Boston University, Boston, Massachusetts, USA.; 7 Department of Radiology, BUSM, Boston, Massachusetts, USA.; 8 Environmental Health Department, School of Public Health, Boston University, Boston, Massachusetts, USA.; 9 Veterans Affairs Boston Healthcare System, Boston, Massachusetts, USA.; 10 Institute of Medical Engineering and Sciences, Massachusetts Institute of Technology, Cambridge, Massachusetts, USA.

**Keywords:** Nephrology, Vascular Biology, Chronic kidney disease

## Abstract

Chronic kidney disease (CKD) imposes a strong and independent risk for peripheral artery disease (PAD). While solutes retained in CKD patients (uremic solutes) inflict vascular damage, their role in PAD remains elusive. Here, we show that the dietary tryptophan-derived uremic solutes including indoxyl sulfate (IS) and kynurenine (Kyn) at concentrations corresponding to those in CKD patients suppress β-catenin in several cell types, including microvascular endothelial cells (ECs), inhibiting Wnt activity and proangiogenic Wnt targets in ECs. Mechanistic probing revealed that these uremic solutes downregulated β-catenin in a manner dependent on serine 33 in its degron motif and through the aryl hydrocarbon receptor (AHR). Hindlimb ischemia in adenine-induced CKD and IS solute–specific mouse models showed diminished β-catenin and VEGF-A in the capillaries and reduced capillary density, which correlated inversely with blood levels of IS and Kyn and AHR activity in ECs. An AHR inhibitor treatment normalized postischemic angiogenic response in CKD mice to a non-CKD level. In a prospective cohort of PAD patients, plasma levels of tryptophan metabolites and plasma’s AHR-inducing activity in ECs significantly increased the risk of future adverse limb events. This work uncovers the tryptophan metabolite/AHR/β-catenin axis as a mediator of microvascular rarefaction in CKD patients and demonstrates its targetability for PAD in CKD models.

## Graphical abstract

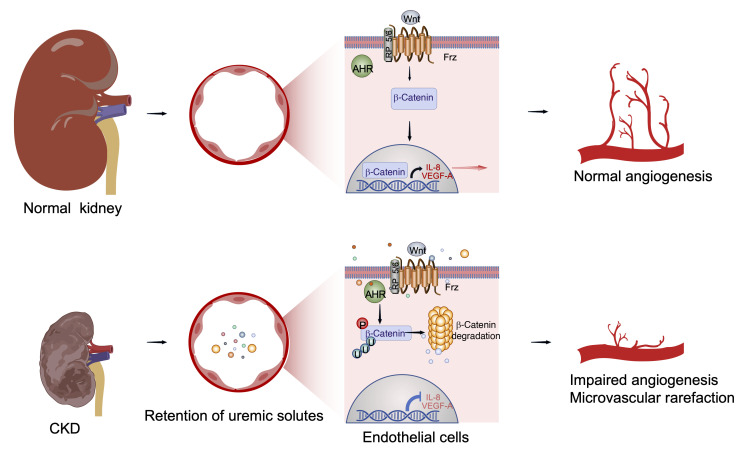

## Introduction

Peripheral artery disease (PAD) is a global health problem affecting 200 million adults worldwide (1, 2). Chronic PAD is characterized by limb hypoperfusion caused by arterial occlusive disease. It can be asymptomatic or can manifest across a spectrum, including intermittent claudication or chronic limb-threatening ischemia (CLTI) (3). CLTI is the end stage of PAD presenting with ischemic rest pain or tissue loss (nonhealing wounds and gangrene), increasing the risk of limb loss. The therapies for PAD are focused on improving limb perfusion to prevent CLTI.

Compared with the general population, patients with chronic kidney disease (CKD) exhibit several unique aspects of PAD (4). Among 1,091,201 patients, those with CKD showed 3 times higher prevalence of PAD (32%) compared with the non-CKD patients (9.6%; ref. 5). The overall 5-year limb-salvage rate in CLTI patients without CKD was 86% and decreased to 50% in patients with stage 5 CKD (6). Even with adjustments for conventional comorbidities, CKD increases the amputation risk by 3-fold (4, 7). Although CKD and non-CKD patients experience a comparable level of arterial patency following revascularization, CKD portends a higher risk for clinical failure defined as amputation and ulceration, etc. (4, 8, 9). These studies indicate CKD as a strong and independent risk factor for PAD and its complications and implicate CKD-specific risk mediators in PAD pathogenesis.

CKD is characterized by the retention of a host of solutes called uremic solutes/toxins (10). A group of uremic solutes are derived from dietary tryptophan (Trp) and include indoxyl sulfate (IS), kynurenine (Kyn), and kynurenic acid (KA), etc. (11). These are known vasculotoxins (12, 13), whose levels increase in the early stages of CKD, rise relentlessly with CKD progression, and are not effectively dialyzed (10). The influence of uremia (a biological state induced by CKD) and these Trp-based uremic toxins on PAD remains poorly explored.

The pathophysiological response to limb ischemia is complex, with a singular goal of compensatory increase in blood flow with the development of macro- and microvasculature (postischemic angiogenesis). This is a dynamic process orchestrated by different signaling processes, of which the Wnt pathway is critical (14). The binding of Wnt ligands such as Wnt3a to Wnt receptors initiates a cascade of events such as inhibition of the glycogen synthetase kinase (GSK-3β), culminating in upregulation and in nuclear translocation of β-catenin (active β-catenin), the central mediator of Wnt signaling (Wnt-on phase; ref. 15). This event induces proproliferative and angiogenic targets, such as cyclin D1, VEGF-A, and IL-8, in endothelial cells (ECs) to increase their proliferation and migration, key processes for angiogenesis. In the absence of a ligand, Wnt signaling is suppressed (Wnt-off phase). Given the central role of Wnt signaling in angiogenesis, we posited that the uremic milieu and Trp-based uremic solutes may downregulate the Wnt/β-catenin pathway in ECs and postischemic angiogenesis.

## Results

### Uremic serum suppresses endothelial functions critical for angiogenesis.

We examined the effect of uremia on functions of human dermal microvascular ECs. The ECs were treated with the pooled sera from 20 end-stage kidney disease (ESKD) patients on hemodialysis (uremic serum). The pooled sera were used first to examine the class effect of CKD. Control sera consisted of age-, sex-, and ethnicity-matched controls without CKD (Supplemental Table 1; supplemental material available online with this article; https://doi.org/10.1172/JCI142260DS1). See complete unedited blots in the supplemental material. ESKD patients showed no differences in age, sex, ethnicity, and blood pressure compared with controls. However, the groups differed in other comorbidities (Supplemental Table 1).

The ECs exposed to uremic serum (final concentration of 5%) showed 30% reduced survival (*P* < 0.001; Supplemental Figure 1A) and a 3-fold lower proliferation (*P* < 0.001) compared with those exposed to control serum (Supplemental Figure 1B). A scratch assay for EC migration showed that ECs exposed to uremic serum migrated half the distance compared with controls (*P* = 0.016; Supplemental Figure 1, C and D).

### Uremic serum suppresses the proangiogenic Wnt/β-catenin axis.

**Figure 1:**
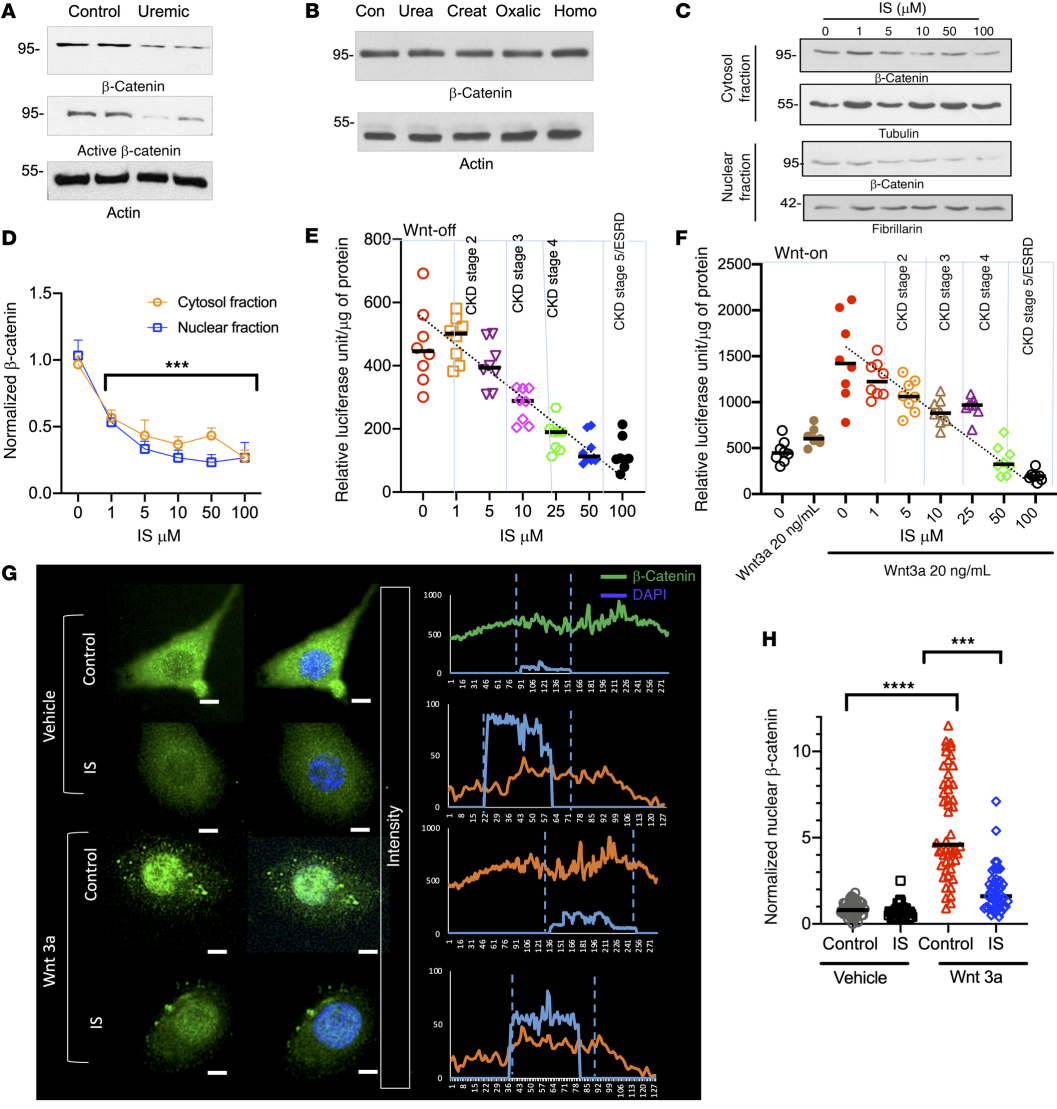
Uremic serum and IS downregulate Wnt/β-catenin signaling in ECs. (**A**). Primary human microvascular ECs exposed to 5% pooled uremic or control sera. Equal amounts of protein were probed for β-catenin and separately for active β-catenin. Actin served as a loading control. Representative images from 3 independent experiments each done in duplicate are shown. (**B**) Representative images of 3 independent experiments of ECs treated with water-soluble uremic toxins. Con, control; Creat, creatinine; Oxalic, oxalic acid; Homo, homocysteine. (**C**). ECs pretreated with 5% of control serum and spiked with IS underwent fractionation. Tubulin and fibrillarin served as markers of fractions and loading controls. Representative images from 4 independent experiments. (**D**) β-Catenin was normalized to loading controls for their fractions. Average of 4 independent experiments is shown. Error bars show SD. The cytosol and nuclear fractions were analyzed separately and compared with the control (IS = 0 μM) using Student’s *t* test with Bonferroni’s correction of multiple comparisons. ***P < 0.001. (**E**) Serum-starved ECs expressing LS were treated with IS-spiked control human serum. Scatter plot of luciferase activity from 2 independent experiments done in quadruplet is shown. The horizontal line in each group corresponds to the median. The blue dotted line corresponds to IS levels in different CKD stages (Supplemental Table 2). A linear regression was performed. (**F**) Luciferase activity assay was performed as above in ECs treated with Wnt3a and IS spiked control human sera. A scatter plot of 2 independent experiments done in quadruplet is shown. A linear regression was performed. (**G**) Serum-starved ECs pretreated with vehicle (PBS + 1% BSA) or Wnt3a (20 ng/mL medium) with IS (50 μM) were stained. Representative images of 100 ECs/group. Profile plots were generated. Note different *y* axis scales between IS-treated and control samples. Scale bars: 5 μm. (**H**) Fifty ECs/group were analyzed. The dot plot represents the integrated density of nuclear β-catenin normalized to the surface area of nucleus. The line corresponds to the median. Independent Student’s *t* tests were performed to compare the groups.***P = 0.003; ****P < 0.001.

The above results suggested that uremic serum suppressed EC functions that are important in angiogenesis. Wnt/β-catenin signaling is critical for EC survival, proliferation, and migration, etc. (14). We posited that the uremic serum might suppress Wnt/β-catenin activity in ECs. ECs treated with uremic sera showed a significant reduction in total β-catenin and active β-catenin compared with controls (both *P* < 0.001; Figure 1A and Supplemental Figure 1E). We assessed Wnt activity in ECs expressing β-catenin–responsive promoter tethered to luciferase reporter construct (LS). The luciferase values were normalized to the protein concentration (relative luciferase unit [RLU]; refs. 16, 17). β-catenin–unresponsive promoter (FuLS) constructs served as a control and showed minimal Wnt activity (Supplemental Figure 1F). Uremic serum suppressed Wnt activity in ECs by more than 10-fold compared with controls. IL-8 and VEGF-A, known Wnt targets in ECs (16, 17), showed 50% reduction in secreted IL-8 (*P* = 0.004) and VEGF-A (*P* = 0.009) compared with controls (Supplemental Figure 1, G and H). Uremic serum reduced cyclin D1, a proproliferative Wnt target induced by Wnt3a (*P* = 0.005; Supplemental Figure 1, I and J), as well as *AXIN2* and *MYC* (Supplemental Figure 1K).

We further probed the factors that may contribute to the Wnt-suppressive effect of uremic serum. Wnt activity in target cells is largely modulated by Wnt ligands, which are glycoproteins (18). We evaluated their involvement using heat-inactivated sera, which is likely to denature proteins. Uremic serum suppressed nuclear β-catenin, and this effect persisted with the heat-inactivated uremic serum (Supplemental Figure 1, L and M), raising the possibility of nonprotein modulators in uremic sera.

### Uremic solutes and Wnt/β-catenin in ECs.

CKD patients retain a host of solutes (nonprotein). Water-soluble solutes such as urea (19.98 mM), creatinine (530 μM), oxalic acid (55.53 μM), and homocysteine (19.97 μM) at concentrations seen in CKD patients had no effect on β-catenin (Figure 1B and Supplemental Figure 1N). In contrast, control sera spiked with IS corresponding to different stages of CKD (Supplemental Table 2) emulated the Wnt-suppressive effect of uremic serum. IS downregulated cytosolic β-catenin by 22% to 30% (*P* = 0.052) and nuclear β-catenin to a greater extent (more than 60%, *P* < 0.001; Figure 1C). In both the fractions, compared with control (IS = 0 μM), IS-treated cells showed approximately 70% to 80% suppression of β-catenin even at concentrations corresponding to those seen in the early stages of CKD (Figure 1D, compared with IS = 0 μM; *P* = 0.004 for IS = 5 μM; *P* < 0.001 for IS = 10, 50, and 100 μM for both the fractions).

We further confirmed these findings on Wnt activity using immunofluorescence (IF) assays. ECs were pretreated with Wnt3a (Wnt-on phase) or vehicle (Wnt-off phase) along with IS (Figure 1, E and F). IS suppressed Wnt activity in a dose-dependent manner (Wnt-off phase, *R^2^* = 0.542 and *P* < 0.001; Wnt-on phase, *R^2^* = 0.768 and *P* < 0.001).

The effect of IS on nuclear β-catenin was analyzed in an IF assay using profile plots. Profile plots were used to examine pixel intensity throughout the length of cells in microns (Figure 1G). IS reduced total β-catenin in ECs by approximately 10-fold (note the differences in the *y* axis scale of Wnt3a- and vehicle-treated cells). The effect on nuclear β-catenin was quantified in randomly selected ECs. Their nuclei were delineated as regions of interest and normalized to the surface area of ECs. This value is presented as the integrated density. Integrated density is a composite of pixel number and intensity and is calculated for the area (μm^2^), as done previously (19, 20). Wnt3a increased nuclear β-catenin by 8- to 10-fold; this was suppressed by IS (Figure 1H, compared with vehicle group, Wnt3a, *P* < 0.001; and compared with Wnt3a, IS plus Wnt-3a group, *P* = 0.003). Collectively, these results suggested that IS downregulated Wnt/β-catenin in ECs.

We assessed whether this effect of IS was EC specific or observed in other cells. In fibroblasts (NIH 3T3) and epithelial cells (human kidney HK-2), IS at 10 μM showed a trend toward increases in cytosolic β-catenin in these cells. However, nuclear β-catenin was consistently downregulated by IS (Supplemental Figure 2, A–D). Skeletal muscle cells were examined due to their involvement in PAD. Treatment of primary human skeletal muscle cells showed that IS monotonically downregulated β-catenin in skeletal muscle cells (Supplemental Figure 2, E and F). These results underscore the broad effect of IS in cell types relevant to PAD.

PAD is characterized by hypoxic environment in the limb. Therefore, we examined IS-mediated β-catenin regulation under a hypoxic environment. ECs exposed to control human serum spiked with different IS concentrations were grown overnight in a hypoxia chamber with 1% O_2_. ECs exposed to 21% O_2_ (normoxia) served as controls. As anticipated, in control samples with IS = 0 μM, nuclear β-catenin levels were higher in hypoxia compared with normoxia (ref. 21 and Supplemental Figure 3, A and B). Under hypoxia, 1 to 10 μM IS suppressed nuclear β-catenin to a greater extent than normoxia, suggesting a more prominent effect of IS on ECs in hypoxic environment. These differences were nonsignificant at higher concentrations of IS.

### Uremic serum and solutes augment β-catenin ubiquitination and degradation.

**Figure 2:**
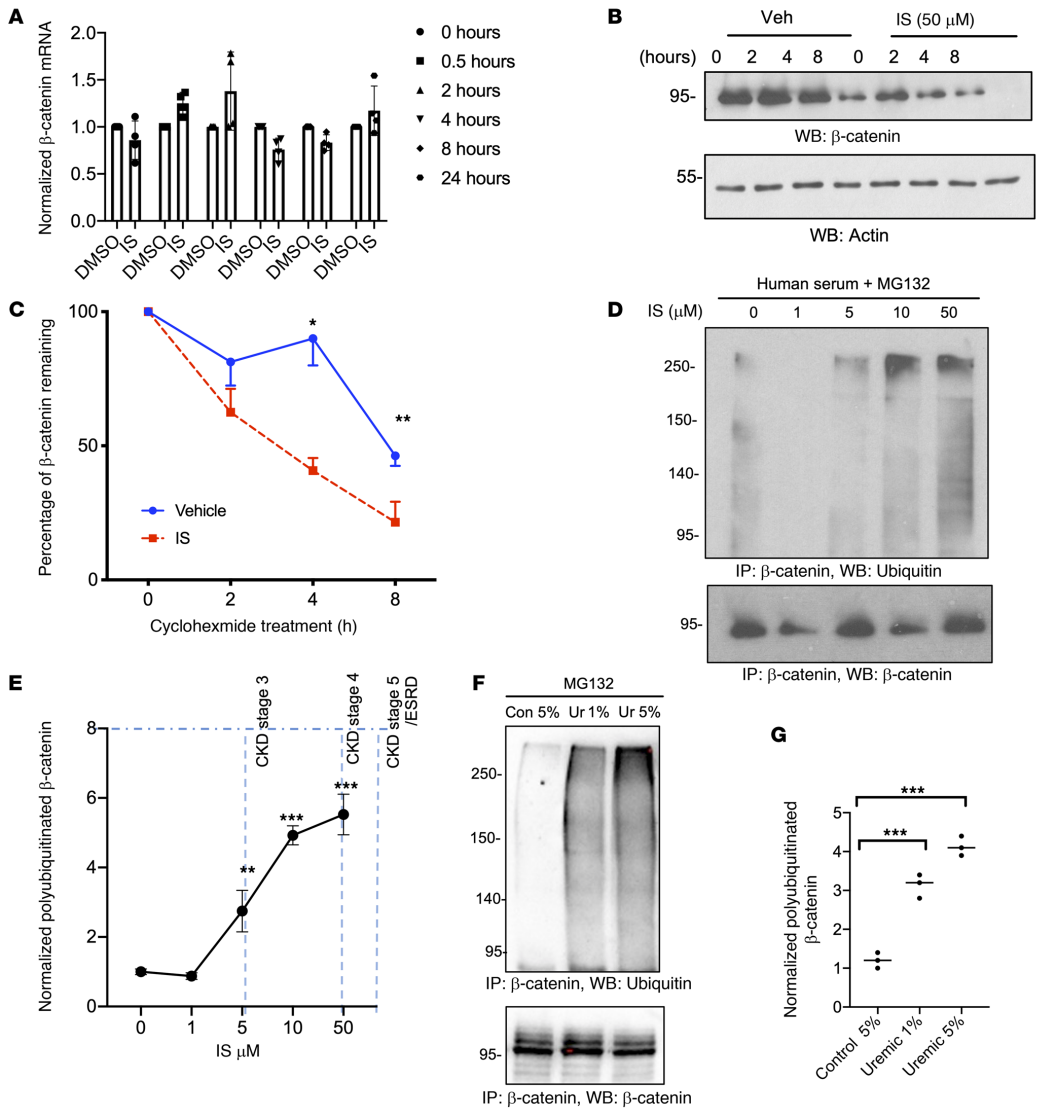
IS augments polyubiquitination and degradation of β-catenin in ECs. (**A**). qRT-PCR analysis of ECs treated with IS 50 μM or DMSO (control) for different times was performed. The average cycle threshold (Ct) values performed in triplicate are shown. Error bars show SD. (**B**). ECs were treated with IS or vehicle (Veh) (DMSO) for 24 hours and cycloheximide (30 μg/mL) for the indicated time. Representative images of 4 independent experiments are shown. (**C**) β-Catenin was normalized to actin and represented as the percentage of remaining β-catenin at each time point. Average of normalized β-catenin from 4 independent experiments is shown. Error bars show SD. Independent Student’s *t* tests were performed. IS treatment at each time point compared with that of vehicle-treated cells. *P = 0.03; **P = 0.001. (**D**) ECs pretreated with 5% control human serum and spiked with IS corresponding to the different human CKD stages. Cells were exposed to 10 μM MG132 overnight. Immunoprecipitation was performed. The blot was reprobed with anti–β-catenin antibody. Representative images of 3 independent experiments are shown. (**E**) Ubiquitinated β-catenin was normalized to immunoprecipitated β-catenin. Average of normalized ubiquitinated β-catenin from 3 experiments is shown. Student’s *t* test with Bonferroni’s correction was performed for multiple comparisons. Error bars show SD. **P = 0.01; ***P < 0.001, compared with vehicle-treated cells. Blue dotted lines show IS levels corresponding to different CKD stages (Supplemental Table 2). (**F**) ECs pretreated with pooled control or uremic sera were processed as above. Blot was reprobed for β-catenin. Representative images of 3 independent experiments are shown. (**G**) Average of normalized ubiquitinated β-catenin from 3 experiments is shown. Independent Student’s *t* tests were performed. Error bars show SD. ***P < 0.001.

IS-induced β-catenin downregulation can be at the transcriptional or posttranscriptional level. ECs treated with IS showed no changes in β-catenin mRNA (Figure 2A), suggesting posttranscriptional β-catenin regulation. β-Catenin is tightly regulated by proteasomal degradation (15), which was determined by a half-life assay. The translation was blocked with cycloheximide, and the amount of time taken for a protein to drop to 50% of its original level was considered its half-life. IS significantly shortened the half-life of β-catenin (control: 7.6 ± 0.32 hours; IS: 2.39 ± 0.46 hours; Figure 2, B and C). We posited that IS might enhance β-catenin polyubiquitination, which was determined by treating cells with MG138, a proteasome inhibitor. β-Catenin ubiquitination was augmented in IS-treated ECs (Figure 2, D and E). ECs treated with increasing concentrations of uremic serum (Figure 2, F and G) showed increased β-catenin ubiquitination by 3- to 4-fold compared with controls (*P* < 0.001). These data suggested that IS and uremic serum augmented β-catenin ubiquitination and degradation in ECs.

### IS regulates β-catenin degradation dependent on serine 33 in the degron motif.

**Figure 3:**
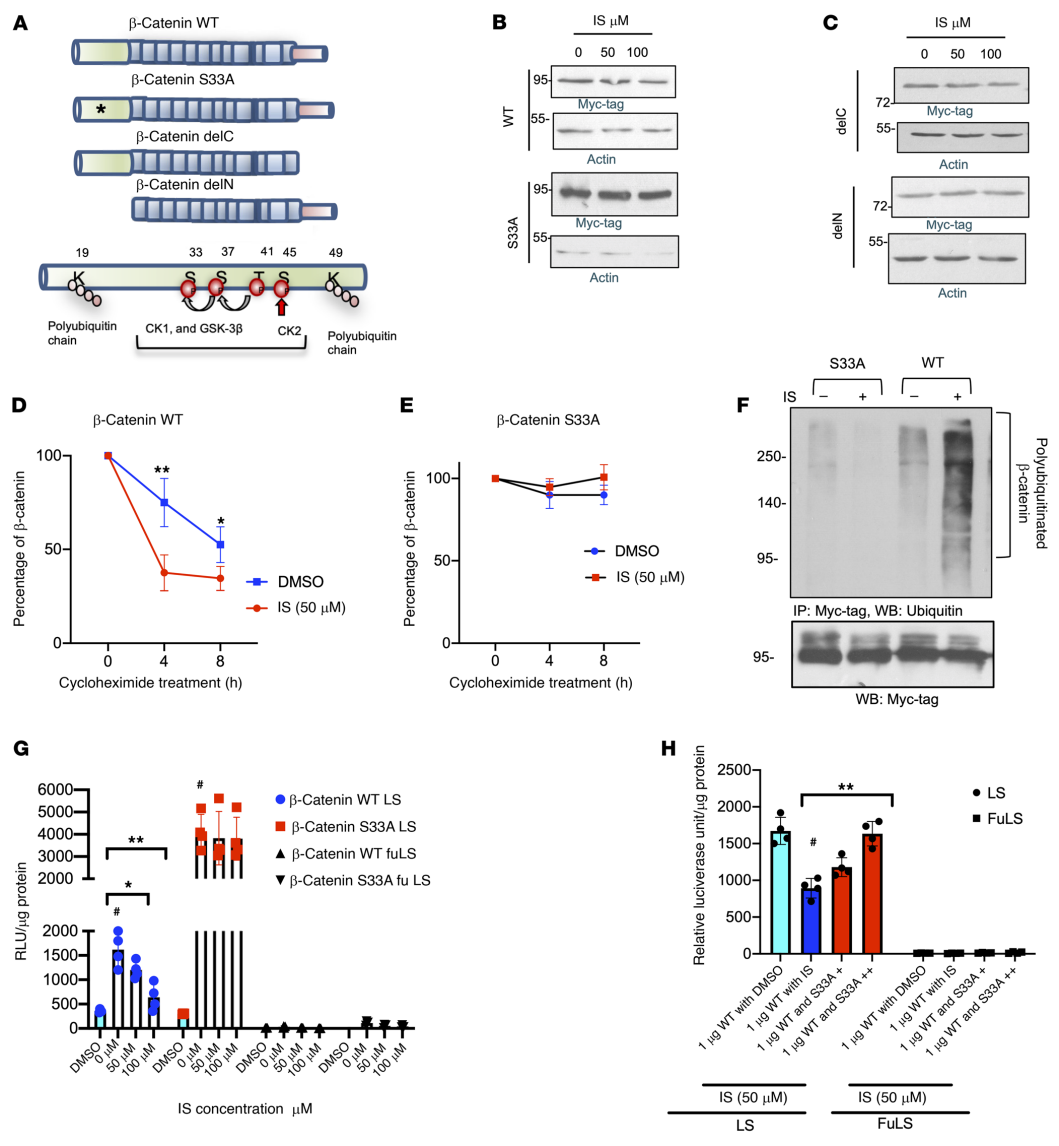
IS downregulates β-catenin dependent on S33 residue in the degron motif. (**A**) β-Catenin N terminus contains a degron motif that controls its degradation. Black asterisk marks β-catenin S33A. Myc-tagged truncations lack N terminus (delN) and C terminus (delC). (**B**) ECs pretransfected with Myc-tagged β-catenin WT or S33A were treated with IS. Representative images of 3 independent experiments. (**C**) ECs pretransfected with Myc-tagged delC or delN β-catenin were treated with IS. Representative images of 3 independent experiments. (**D**) ECs transfected with WT β-catenin were treated with IS (50 μM) and cycloheximide (30 μg/mL) for indicated times. Average of normalized β-catenin from 4 independent experiments is shown. Error bars show SD. Independent Student’s *t* tests were performed at different time points. **P = 0.01; *P = 0.04. (**E**) Half-life study of Myc-tagged β-catenin S33A was performed as above from 4 independent experiments. Error bars show SD. (**F**) ECs pretransfected with Myc-tag β-catenin WT or S33A were treated with 50 μM IS and 10 μM MG132 before harvest. Immunoprecipitation was performed. Lysates were probed separately with anti-Myc-tag antibody. Representative images of 3 independent experiments are shown. (**G**) ECs stably expressing LS and Fu LS constructs were cotransfected with Myc-tagged WT or S33A β-catenin and were treated with IS or DMSO followed by a luciferase assay. Average of 3 experiments done in triplicate is shown. Error bars show SD. Independent Student’s *t* tests were performed. *P = 0.043; **P = 0.003; ^#^*P* <0.0001. (**H**) ECs stably expressing LS and FuLS constructs were cotransfected with Myc-tagged WT and increasing amounts of S33A β-catenin (shown as + and ++ marks). Expression of β-catenin WT or S33A was confirmed (Supplemental Figure 5E). Average of 3 experiments done in triplicate is shown. Error bars show SD. Independent Student’s *t* tests were performed. **P = 0.002; ^#^*P* = 0.001.

β-Catenin N terminus contains a “degron” motif that regulates its protein stability. This motif is characterized by a series of serine and threonine, which in Wnt-off phase undergoes sequential phosphorylation by casine kinases (CK1 and CKD2) and GSK-3β (Figure 3A and ref. 22). The phosphorylation of serine 33 signals ubiquitinates of flanking lysine residues degrading β-catenin (15). Wnt activation (Wnt-on phase) suppresses GSK-3β, and β-catenin undergoes nuclear translocation. We investigated IS-induced β-catenin regulation by generating a truncation of β-catenin lacking the degron motif (delN, lacking the N terminus of β-catenin) or by introducing a point mutation at serine 33 (S33A) that renders β-catenin stable and transcriptionally active. Also, truncation of C terminus (delC) was created with the intact degron motif (Figure 3A).

ECs expressing these Myc-tagged constructs (Supplemental Figure 4A) were treated with IS. IS suppressed WT and delC β-catenin, while β-catenin S33A and delN remained resistant to IS (Figure 3, B and C, and Supplemental Figure 4B). This effect of IS corroborated with the IF and half-life studies. WT β-catenin expressed predominantly in the nuclei was downregulated by IS (Supplemental Figure 4C). β-Catenin S33A was expressed in the cytosol and nuclei of ECs and showed no changes with IS. Myc-tagged WT β-catenin reached 50% of its original level by 8 hours, which was shortened to 4 hours by IS. The half-life of β-catenin S33A was unaltered by IS (Figure 3, D and E, and Supplemental Figure 4D). IS increased the polyubiquitination of WT β-catenin by 2-fold (Figure 3F and Supplemental Figure 5A), but did not increase that of S33A β-catenin. These results suggested that IS augmented β-catenin degradation dependent on serine 33 in the degron motif.

We next examined the effects of IS on Wnt activity and Wnt targets in response to these constructs. WT or S33A β-catenin increased Wnt activity above baseline (Figure 3G, β-catenin WT or S33A increased Wnt activity from DMSO control, *P* < 0.0001; in the IS-treated group, compared with IS = 0 μM, *P* = 0.043 and *P* = 0.003). IS suppressed Wnt activity and secreted IL-8 and VEGF-A in EC media expressing WT β-catenin, but not β-catenin S33A (Supplemental Figure 5, B and C). Finally, we posited that β-catenin S33A is likely to rescue IS-mediated Wnt suppression. ECs expressing LS constructs and Flag-tagged WT β-catenin were cotransfected with increasing amounts of S33A β-catenin (Figure 3H and Supplemental Figure 5E). IS significantly suppressed Wnt activity induced by WT β-catenin (Figure 3H). In WT-transfected cells, compared with control (marked as DMSO), IS suppressed Wnt activity (*P* = 0.001). A combination of 0.4 μg of S33A along with β-catenin WT activity increased Wnt activity compared with β-catenin WT alone (*P* = 0.002). No difference in Wnt activity was noted between β-catenin WT with DMSO and 0.4 μg of S33A plus β-catenin WT treated with IS, suggesting that β-catenin S33A restored Wnt activity to baseline in IS-treated ECs. The levels of IL-8 and VEGF-A followed Wnt activity (Supplemental Figure 5D).

### A set of Trp metabolites regulate Wnt/β-catenin signaling.

We determined whether this effect of IS is shared by other Trp metabolites that are retained in CKD patients (Supplemental Table 2). Dietary Trp is processed by the gut microbiome to indole, which is subsequently converted to IS and indole-3 acetic acid (IA) in liver. Trp absorbed from gut is converted to Kyn, anthranilic acid (AA), KA, xanthurenic acid (XA), and quinolinic acid (QA) (Supplemental Figure 6). These are elevated in patients beginning with the early stages of CKD, and their levels vary in patients at the same stage of CKD (refs. 23–26 and Supplemental Table 2) because their levels are influenced by dietary intake of Trp and processing by intestinal microbiome, etc. ECs expressing LS were exposed to these solutes at concentrations observed in CKD patients in the absence (Wnt-off phase) or presence of lithium chloride, a known inhibitor of GSK-3β and Wnt activator (Wnt-on phase; refs. 27, 28 and Supplemental Figure 7A). Lithium chloride significantly upregulated Wnt signaling. Kyn, KA, and XA significantly inhibited Wnt activity in a dose-dependent manner (Supplemental Figure 7, B–H). AA or QA had no effect on Wnt activity in the Wnt-on phase. We confirmed the effect of Kyn on β-catenin. ECs treated with Kyn corresponding to different stages of CKD (Supplemental Table 2) significantly suppressed nuclear β-catenin (Supplemental Figure 8, A and B) and augmented β-catenin ubiquitination even at concentrations observed in the early stages of CKD (Supplemental Figure 8, C and D). These data suggested that several Trp metabolites suppressed Wnt/β-catenin activity in ECs, pointing to a common mechanism of Wnt/β-catenin suppression.

### Uremic serum and solutes suppress Wnt/β-catenin signaling through AHR.

IS, Kyn, KA, and XA are activators of the aryl hydrocarbon receptor (AHR) pathway in various cells (12, 23, 24, 29). Therefore, we posited that uremic serum and these Trp metabolites regulated Wnt/β-catenin signaling through AHR, which was examined using its genomic and pharmacological manipulation.

**Figure 4:**
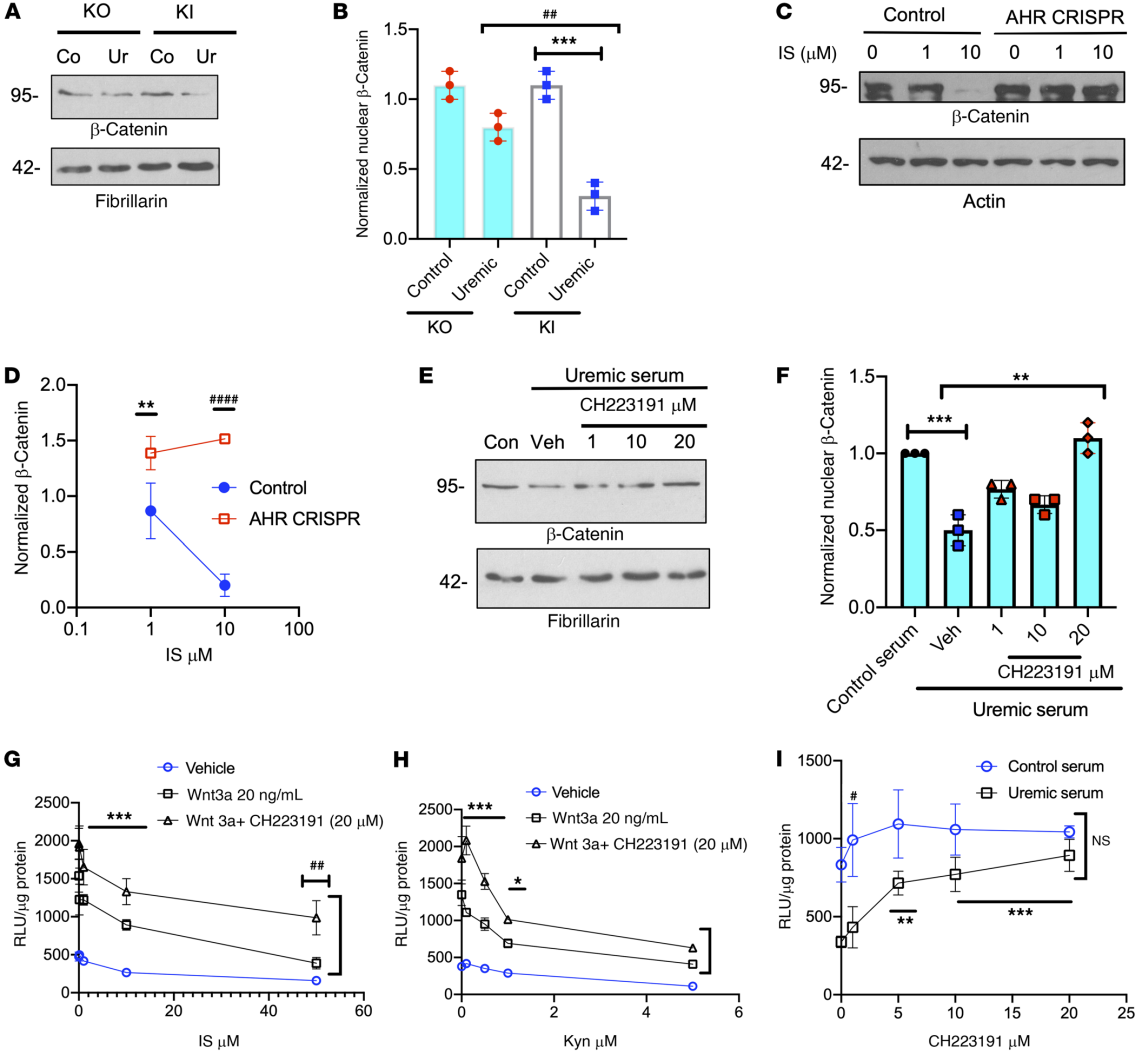
Uremic serum and uremic solutes mediate Wnt/β-catenin suppressive effect through AHR signaling. (**A**) AHR KI and KO MEFs treated with pooled 5% control or uremic sera. Their nuclear fractions were probed. Representative images of 3 independent experiments are shown. (**B**) Average of normalized nuclear β-catenin from 3 experiments is shown. Independent Student’s *t* tests were performed. Error bars show SD. ^##^*P* = 0.032; ***P = 0.006. (**C**) ECs knocked out of AHR (AHR CRISPR) were treated with IS. Representative images of 3 independent experiments are shown. (**D**) Average of normalized β-catenin from 3 independent experiments. Bars show SEM. Student’s *t* test was performed. **P = 0.037; ^###^*P* < 0.001. (**E**) Representative images of nuclear fractions of ECs treated with 5% pooled uremic serum with CH223191 from 3 independent experiments are shown. (**F**) Average of normalized β-catenin from 3 experiments is shown. Student’s *t* test with Bonferroni’s correction was performed for multiple comparisons. Error bars show SD. **P = 0.004; ***P < 0.001. (**G**–**I**) ECs expressing LS were treated with IS and 20 ng/mL Wnt3a with or without 20 μM CH223191 (**G**), with Kyn and Wnt3a 20 ng/mL with or without 20 μM CH223191 (**H**), with 5% pooled control or uremic serum with CH223191. ***P = 0.01; ^##^*P* = 0.041 (**G**). ***P = 0.001, *P = 0.025 (**H**). (**I**) Averages for 6 independent repeats in each figure are shown. Error bars show SEM. ANOVA test was performed to compare groups (*P* < 0.001). ^#^*P* = 0.01; **P = 0.01; ***P = 0.001. Pairwise comparisons with Tukey’s multiple comparison procedure were performed.

We examined mesenchymal embryonic fibroblasts (MEFs) from AHR KO mice and compared them with MEFs with restored AHR expression (knockin [KI] cells; ref. 24 and Figure 4, A and B). Uremic serum induced approximately 80% downregulation of nuclear β-catenin in the AHR KI MEFs (*P* < 0.001) compared with controls, while AHR KO MEFs showed a nonsignificant trend toward reduction in nuclear β-catenin with uremic serum. Importantly, an approximately 2.4-fold reduction in nuclear β-catenin (*P* = 0.032; Figure 4B) was observed with uremic serum–treated AHR KI MEFs compared with AHR KO MEFs. These findings were validated in ECs using the AHR CRISPR technique (Supplemental Figure 9A), which resulted in substantial downregulation of AHR protein compared with that in control ECs (Supplemental Figure 9B). AHR CRISPR ECs showed an approximately 35% to 55% increase in β-catenin compared with control cells (*P* < 0.001; Supplemental Figure 9, C and D). The uremic serum-treated control cells showed 60%–70% downregulation of β-catenin compared with the control serum–treated cells (*P* = 0.006). This effect of uremic serum was abrogated in AHR CRISPR cells (Supplemental Figure 9, E and F). Uremic serum–treated AHR CRISPR ECs showed an approximately 3.2-fold increase in β-catenin levels compared with control ECs (*P* = 0.003). A similar pattern was observed in AHR CRISPR ECs with IS (Figure 4C). IS downregulated β-catenin, which was abrogated in AHR CRISPR ECs (Figure 4D, compared with control cells, AHR CRISPR cells, *P* = 0.037 for IS = 1 μM and *P* < 0.001 for 10 μM). These results suggested that AHR activity in ECs is necessary for IS- and uremic serum–induced Wnt/β-catenin suppression.

CH223191, a specific AHR inhibitor (24) abrogated β-catenin suppression with uremic serum (Figure 4E). Uremic serum suppressed β-catenin (Figure 4F, P < 0.001). Compared with uremic-treated control cells, ECs treated with uremic serum plus 20 μM CH223191 showed a 2-fold upregulation of β-catenin (*P* = 0.004). AHR inhibition restored IS-induced suppression of Wnt activity (Figure 4G). IS suppressed Wnt activity in the presence of Wnt3a or vehicle. However, cotreatment of IS plus 20 μM CH223191 upregulated Wnt activity (compared with ECs treated with Wnt3a plus IS, those with Wnt3a plus IS plus CH223191 increased Wnt activity, *P* = 0.01 [compares Wnt3a plus IS versus Wnt3a plus IS plus CH223191 for the indicated concentrations of IS], *P* = 0.041 [compares Wnt3a plus IS versus Wnt3a plus IS plus CH223191 at IS of 50 μM]). A similar increase in Wnt activity was noted with Kyn in its highest concentration (5 μM; Figure 4H). Compared with ECs treated with Wnt3a plus Kyn, those cotreated with Wnt3a plus Kyn plus CH223191 showed higher Wnt activity (*P* = 0.001 [compares Wnt3a plus Kyn versus Wnt3a plus Kyn plus CH223191 for the indicated concentrations of IS], *P* = 0.025 [compares Wnt3a plus IS versus Wnt3a plus Kyn plus CH223191 at IS of 5 μM]; Figure 4H).

Finally, we assessed Wnt activity with increasing concentrations of CH223191. ECs expressing the LS construct were treated with 5% pooled uremic serum, which suppressed Wnt activity compared with control serum (noted as*P* = 0.01 in Figure 4I). Compared with the uremic serum alone, cotreatment of uremic serum plus CH223191 increased Wnt activity in ECs (*P* = 0.01 at 5 μM; *P* = 0.001 for 10 and 20 μM). In fact, there was no significant (Figure 4I) difference in the Wnt activity between control-serum treated cells and uremic serum plus CH223191–treated cells. These results suggested that AHR inhibitor restored Wnt suppression in the CKD milieu to the level of non-CKD.

### IS reduces vasculogenesis in zebrafish.

We probed the effect of IS in a Fli1-eGFP transgenic zebrafish model, a well-established model of de novo angiogenesis (vasculogenesis). In this model, ECs are genetically engineered to express EGFP (17). The thickness and bifurcation of intersegmental vessel (ISV) and tail microvasculature are biological readouts. Zebrafish embryos observed at 24 hours post fertilization (hpf) were treated with IS for an additional 48 hours. Zebrafish exposed to IS showed reduced ISV length and thickness and reduced bifurcation of ISVs (white and blue arrowheads, Supplemental Figure 10, A and B). Compared with DMSO controls, IS at 50 μM suppressed the tail microvasculature by 47% (*P* = 0.012) and by 90% at 100 μM (*P* = 0.002; Supplemental Figure 10, C and D). This model demonstrated the suppressive effect of IS on vasculogenesis.

### IS suppressed Wnt/β-catenin axis in the capillaries and postischemic angiogenesis in mice.

**Figure 5:**
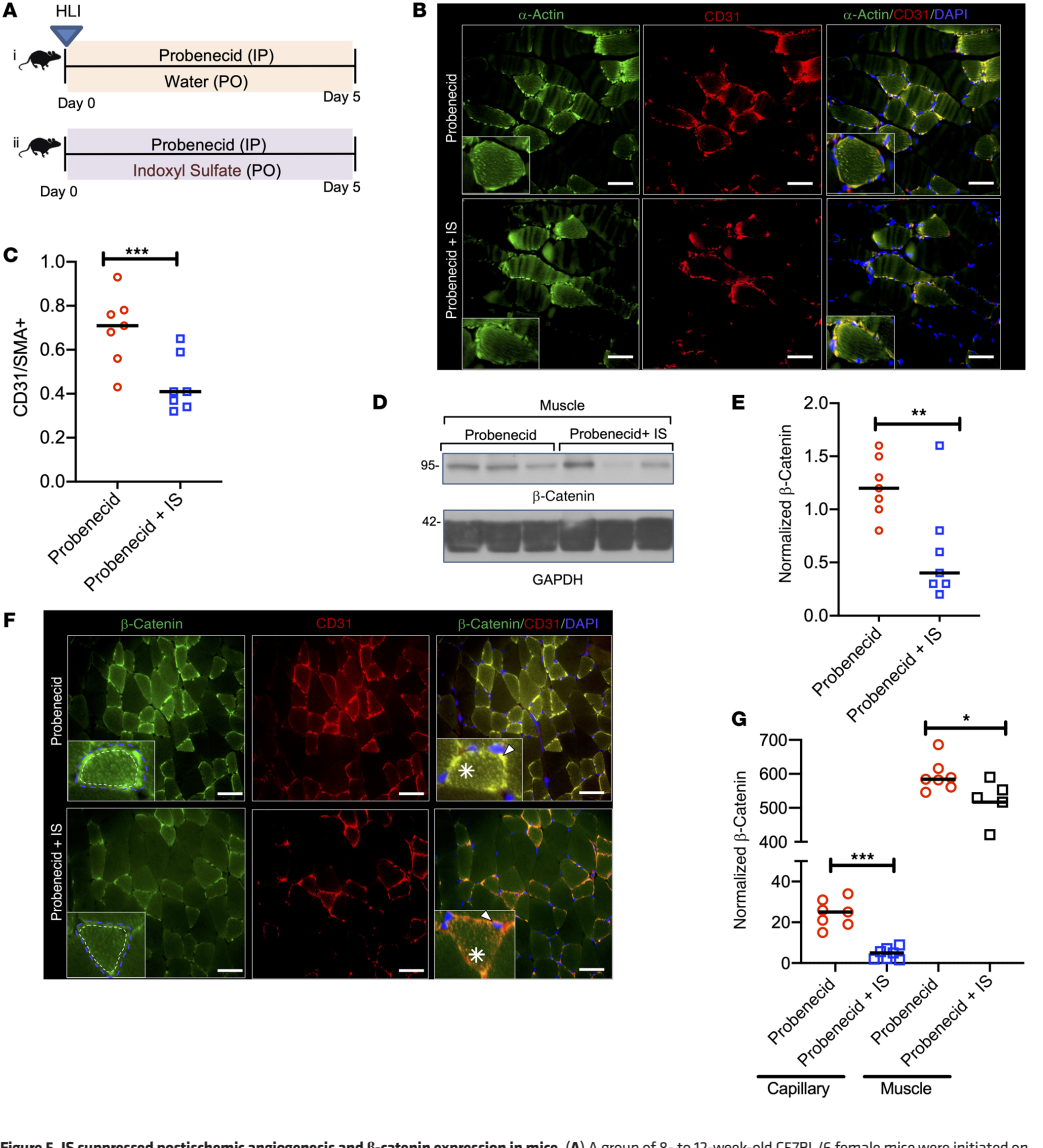
IS suppressed postischemic angiogenesis and β-catenin expression in mice. (**A**) A group of 8- to 12-week-old C57BL/6 female mice were initiated on probenecid (*n* = 7) or probenecid plus IS (*n* = 7) and underwent HLI followed by harvest after 5 days. (**B**) Representative images of the posterior calf muscles from the ligated limbs of mice stained for α-actin and CD31. Total of 30 images per group (*n* = 7 mice/group). Insets show myocyte with surrounding capillaries. Scale bars: 25 μm. Original magnification ×400. (**C**) Integrated density of CD31^+^ was normalized to that of α-actin and presented as a ratio. A total of 30 images from 7 mice/group are represented, and the line represents the median. Student’s *t* test was performed. ***P = 0.001. (**D**) Lysates of posterior calf muscles of the ligated limb of mice were probed. Representative immunoblots from 3 separate mice from each group (*n* = 7 mice/group). (**E**) Normalized β-catenin in muscle lysates is presented in box-and-whisker plot. Student’s *t* test was performed. **P = 0.007. (**F**) Four to five random images of posterior calf muscles from the ligated limbs of mice were stained for β-catenin and CD31. Insets show a myocyte with β-catenin with surrounding capillaries. Blue dotted line represents ROI of a muscle surrounded by capillaries, and white dotted line represents the ROI of a muscle. White asterisk corresponds to β-catenin in a muscle. White arrowhead is directed to β-catenin in the capillary. Scale bars: 25 μm. Original magnification, ×400. (**G**) First, the integrated density was estimated from the ROI of area surrounded by blue dotted line (β-catenin in a muscle with surrounding capillary). Next, the integrated density was analyzed only from area surrounded by white dotted line (β-catenin in muscles). Their differences correspond to β-catenin in the capillaries. The integrated densities of capillaries and muscles from 30 random images (*n* = 7 mice/group) are shown. Lines represent the median. Student’s *t* test was performed. ***P < 0.001; *P = 0.05.

Human PAD is induced in responses to arterial occlusion, and this pathology is not well emulated in the zebrafish model. We investigated the effect of IS in a hindlimb ischemia (HLI) model, which is a widely used murine model of PAD. Mice exposed to IS (30, 31) underwent HLI (Figure 5A). In this model, the mice were administered IS in drinking water and its excretion was suppressed by probenecid, raising blood levels of IS corresponding to those in advanced CKD patients (30, 31). Mice treated with probenecid alone served as controls. The blood urea nitrogen (BUN) levels of mice were comparable between both groups, suggesting their normal renal function (Supplemental Figure 11A). Serum IS levels were approximately 12-fold higher in IS-administered mice compared with the control (*P* < 0.001; Supplemental Figure 11B). This model examined the effect of higher levels of IS over a short time frame.

Capillary density was examined using CD31, a marker of ECs, and normalized to muscles stained with α-actin (Figure 5B). Expression of both these proteins on IF was examined in randomly obtained images of muscles in each mouse and was quantitated as the integrated density, as done previously (19, 20). Compared with the integrated density of CD31/SMA^+^ for probenecid-exposed controls, an approximately 30% reduction was observed in IS-exposed mice (*P* = 0.0014; Figure 5C).

**Figure 6:**
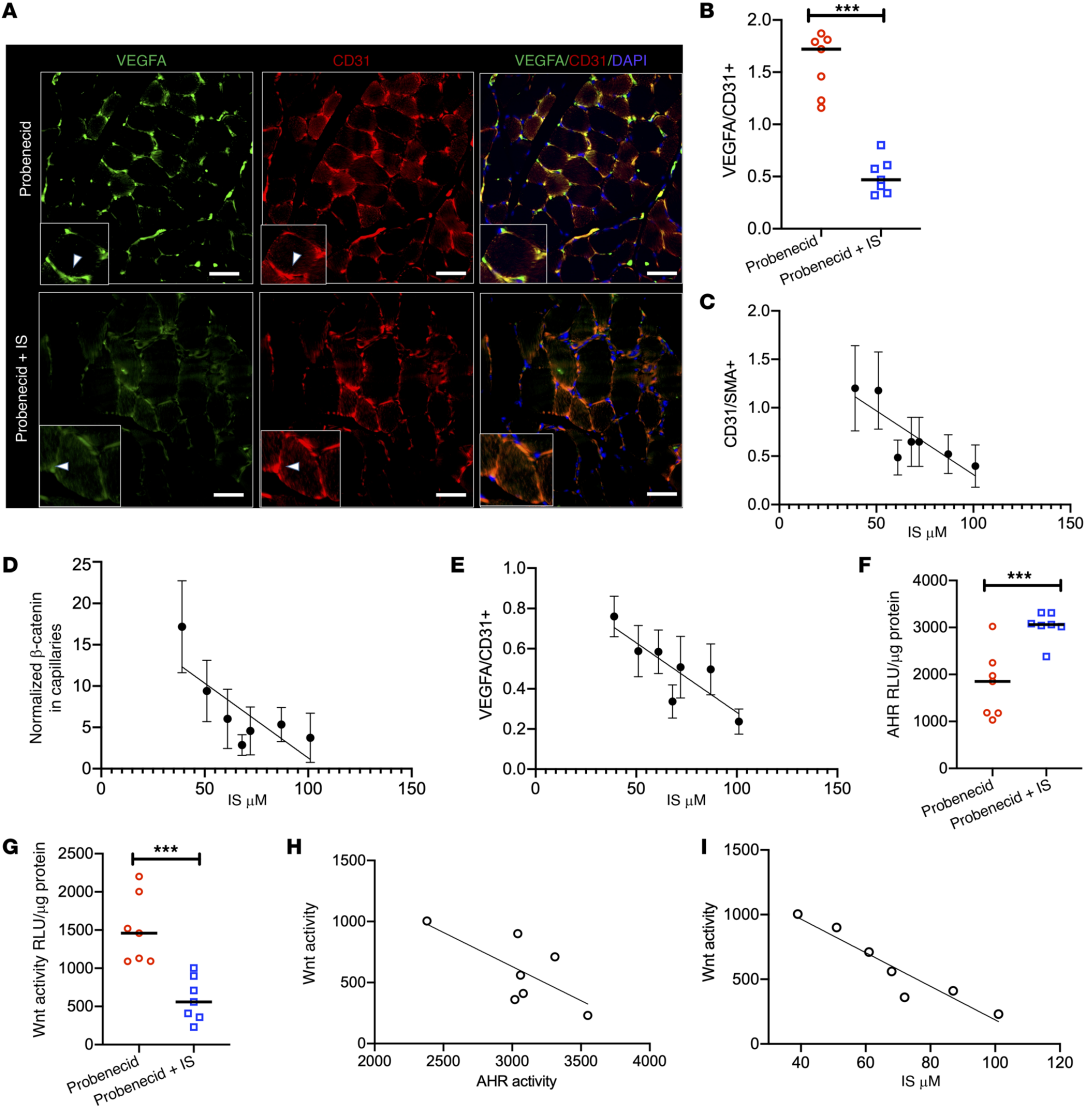
IS-specific solute mouse model shows suppressed VEGF-A expression and angiogenic phenotype in an IS- and AHR-dependent manner. (**A**) Representative from 30 random images (*n* = 7 mice/group) of the posterior calf muscles from the ligated limbs were stained. Insets show a representative myocyte with surrounding capillaries. White arrowheads correspond to VEGF or CD31. Scale bars: 25 μm. Original magnification, ×400. (**B**) Integrated density of VEGF-A and CD31 were estimated from 30 images from each group (*n* = 7 mice/group). Lines represent the median. Student’s *t* test was performed. ***P = 0.001. (**C**–**E**) Correlations between IS levels and capillary density (CD31/SMA^+^) (**C**) and normalized β-catenin in capillaries (**D**) and normalized VEGF-A in the ischemic limb of IS-exposed mice (*n* = 7) (**E**). (**F** and **G**) ECs expressing XRE-responsive promoter (**F**) or LS (**G**) treated with sera from mice from 2 groups (*n* = 7/group). Lines correspond to median. Student’s *t* test was performed. ***P < 0.001. (**H** and **I**) Correlation between EC Wnt and AHR activity in response to sera from IS-exposed mice (**H**) and IS levels and EC Wnt activity in response to sera from these mice *n* = 7 (**I**).

The β-catenin levels were examined in the lysates of posterior calf muscles from the ligated limbs. The CKD mice showed an approximately 1.5-fold lower β-catenin than the controls (*P* = 0.034; Figure 5, D and E). This reduction in β-catenin can be from capillaries and/or skeletal muscles. We observed β-catenin reduction in both the capillaries and skeletal muscles in IS-treated mice compared with control mice (Figure 5F). We further quantitated reduction in both these cell types. First, a region of interest (ROI) involved the muscle fiber along with the capillaries (Figure 5F) and its integrated density was noted. A similar analysis was performed by delineating muscle alone (Figure 5F). The difference between these 2 values provided the β-catenin levels in capillaries. β-Catenin expression in muscle was reduced by 10% to 12% in IS-treated mice compared with control mice (*P* = 0.054; Figure 5, F and G). A greater reduction in β-catenin was observed in capillaries (4-fold) in IS-treated mice compared with the control mice (*P* < 0.001; Figure 5 F and G). VEGF-A expression was exclusively observed in the capillaries, as identified by colocalization with CD31 (Figure 6A). As capillary density is reduced in IS-treated muscles, it can affect the evaluation of VEGF-A expression, and therefore, we normalized VEGF-A expression to CD31. IS-treated muscles showed an approximately 50% reduction in normalized VEGF-A expression (*P* = 0.001; Figure 6B).

To further establish the role of IS in postischemic angiogenesis, we performed correlation analyses of the serum levels of IS in mice (obtained at the end of the experiment) with the features of postischemia angiogenesis. The levels of IS correlated inversely with the capillary density (*R^2^* = 0.499, *P* < 0.001), β-catenin levels in the capillaries (*R^2^* = 0.405, *P* < 0.001), and VEGF-A expression (*R^2^* = 0.518, *P* < 0.001; Figure 6, C–E).

Next, we investigated relationships between AHR and Wnt activities in ECs in response to sera from mice exposed to IS. Sera from mice at a final concentration of 5% was used to treat ECs stably expressing LS construct (Wnt activity) or the xenobiotic-responsive element (XRE) promoter tethered to the luciferase reporter (to measure AHR activity). A 3-fold downregulation in EC Wnt activity (*P* < 0.001) and approximately 2-fold upregulation of AHR activity (*P* = 0.002) were observed in response to sera from mice exposed to IS compared with the control group (Figure 6, F and G). AHR activity in the ECs negatively correlated with Wnt activity (*R^2^* = 0.518, *P* < 0.001; Figure 6H). Importantly, IS levels in the blood of the CKD mice inversely correlated with EC Wnt activity (*R^2^* = 0.897, *P* < 0.001; Figure 6I). These results demonstrated that short exposure to IS at high levels significantly downregulated β-catenin and VEGF-A expression in capillaries and reduced capillary density.

### CKD milieu suppresses postischemic angiogenesis and Wnt/β-catenin axis in capillaries and muscles.

**Figure 7:**
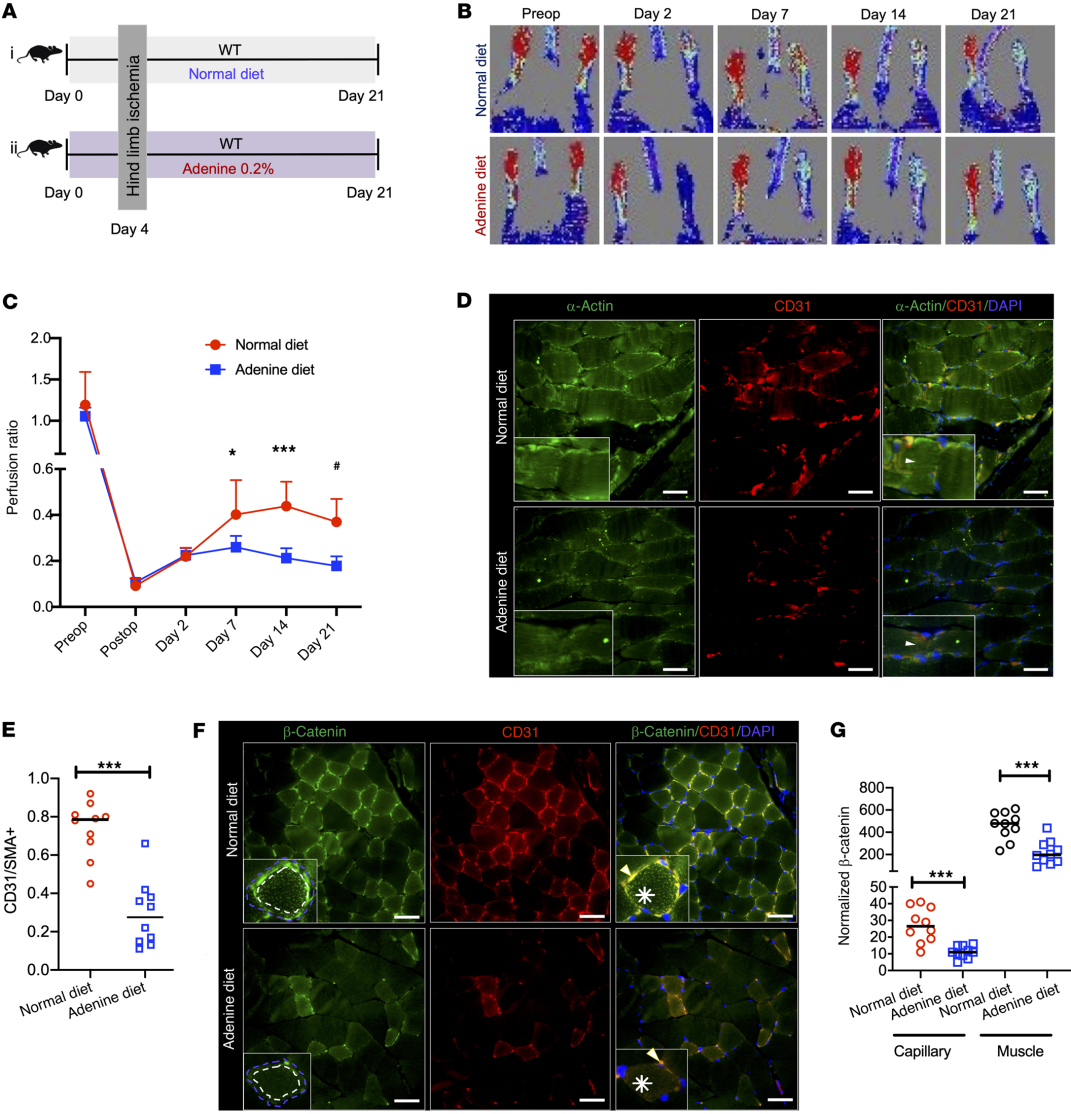
Adenine-induced CKD model shows compromised angiogenesis and β-catenin expression in the ligated limb of mice. (**A**) A group of 8- to 12-week-old C57BL/6 female mice on a 0.2% adenine diet (*n* = 10) or the control diet (*n* = 10) underwent HLI and mice were harvested at the end of 21 days. (**B**) Representative laser doppler images of mouse hind paws. *n* = 10 mice/per group. (**C**) Average perfusion ratios. Error bars show SD. Independent Student’s *t* tests were applied to compare 2 groups at each time point. *P = 0.05; ***P < 0.001; ^#^*P* = 0.002. (**D**) Three random images from stained posterior calf muscles of the ligated limbs of each mouse (*n* = 10 mice/group). Insets represent myocytes, where white arrowheads are directed at α-actin expression. Scale bars: 25 μm. Original magnification ×400. (**E**) Averages of the normalized integrated density of CD3 to α-actin per image described in Figure 6D are shown. Lines represent median. Student’s *t* test was performed. ***P < 0.001. (**F**) Three random images of stained posterior calf muscles of the ligated limb per mouse (*n* = 10 mice/group). Insets show a myocyte stained with β-catenin with surrounding capillaries. Blue dotted lines represent ROI of a myocyte and capillaries; white dotted lines represent ROI of a myocyte. White asterisks correspond to β-catenin in a myocyte. White arrowheads are directed to β-catenin in a capillary. Scale bars: 25 μm. Original magnification ×400. (**G**) Normalized integrated densities of β-catenin of muscles and capillaries obtained from 30 random images per group (*n* = 10 mice/group). Lines represent median. Student’s *t* test was performed. For capillary ***P < 0.001 and for muscle ***P = 0.001.

Next, we examined the postischemic angiogenesis and role of AHR signaling in a CKD model. We used an adenine-induced CKD mouse model, which is characterized by high blood levels of IS and Kyn (30, 31), and recapitulated several uremic manifestations (30, 32). A group of 10 C57BL/6 mice were exposed to a 0.2% adenine diet and underwent HLI on day 4 after the adenine diet (Figure 7A). Mice on a normal diet served as controls, and all of them were followed for a total of 21 days before the harvest. Kidneys of adenine-exposed mice showed glomerulosclerosis, tubular dilatation, and interstitial fibrosis (Supplemental Figure 12A) and had a significant increase in interstitial fibrosis and tubular atrophy (IFTA) score (ref. 33 and Supplemental Figure 12B). Compared with the control mice, adenine-treated mice showed increases in BUN and creatinine (Supplemental Figure 12, C and D) and a 7-fold increase in serum IS levels (*P* < 0.001; Supplemental Figure 12E).

Capillary perfusion recovery was followed over 21 days using a laser doppler and presented as a ratio of perfusion in the ligated limb to the unligated limb (Figure 7B). On postoperative day 7, the perfusion ratio of mice on the adenine diet was half of that of the control mice on a normal diet (*P* = 0.05) and remained reduced at 14 (*P* < 0.001) and 21 days after HLI (*P* = 0.002) (Figure 7C). Consistent with the above findings, the muscles of ligated limbs of adenine-exposed mice showed approximately 50% reduced capillaries (*P* < 0.001; Figure 7, D and E).

**Figure 8:**
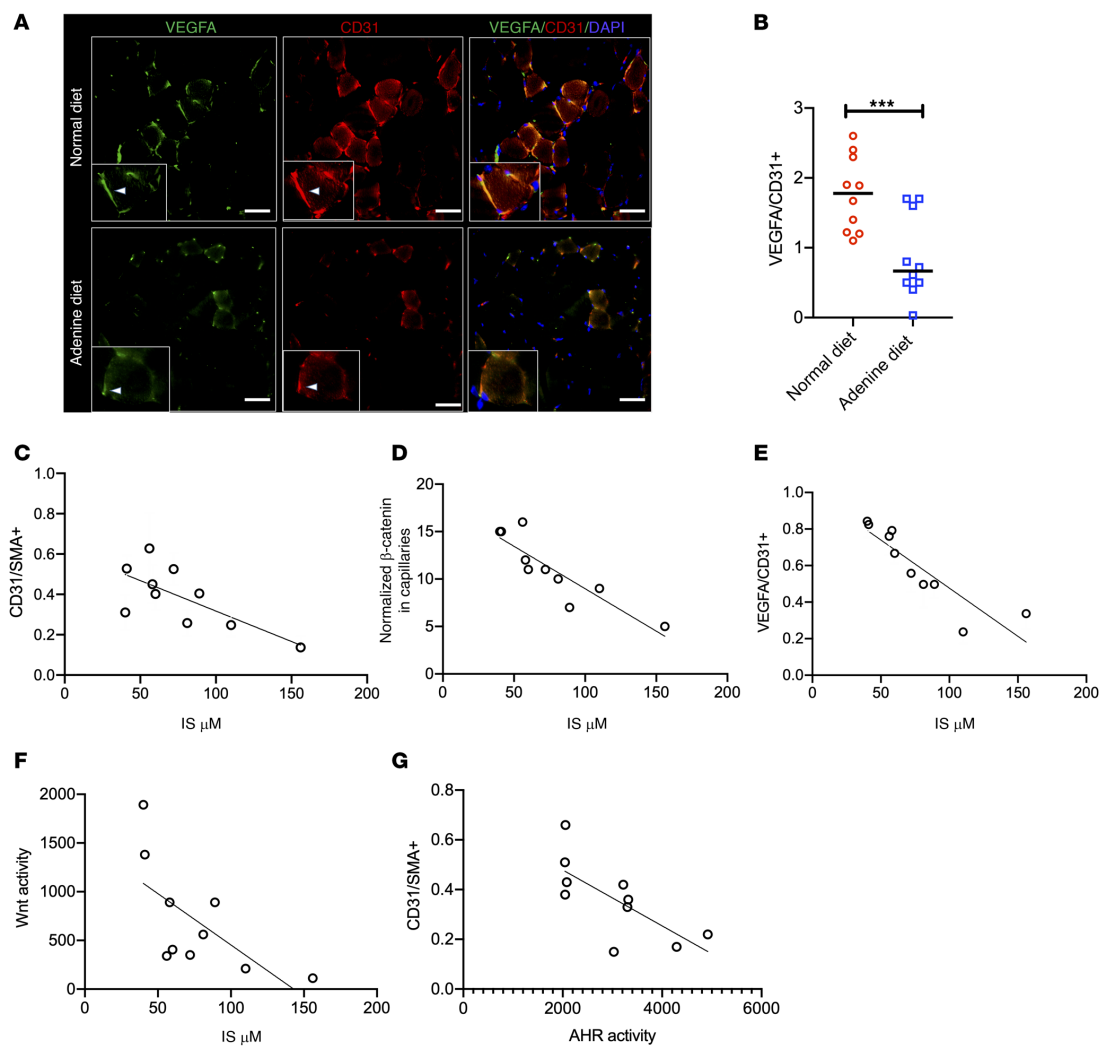
Adenine-induced CKD model shows suppressed VEGF-A and angiogenic response in an IS- and AHR-dependent manner. (**A**) Representative images from 3 randomly taken images per mouse (*n* = 10 mice/group). Insets show representative myocytes with white arrowheads directed to VEGF or CD31. Scale bars: 25 μm. Original magnification ×400. (**B**) The integrated densities of normalized VEGF-A from images obtained from Figure 6H. Lines represent median. Student’s *t* test was performed. ***P = 0.003. (**C**–**E**) Correlations between IS levels and histological parameter in the ischemic limb of CKD mice (*n* = 10). (**F** and **G**) Correlations between EC Wnt activity in response to sera from CKD mice and their IS levels (**F**) and the capillary density in CKD mice and AHR activity in ECs in response to sera from these mice (**G**). *n* = 10.

The mice on an adenine diet showed 80% reduction in β-catenin protein compared with the control (*P* < 0.001; Supplemental Figure 12, F and G). Our IF studies demonstrated an approximately 40% reduction of β-catenin in skeletal muscles and a greater reduction in β-catenin (90%) in capillaries in adenine mice compared with the control mice (Figure 7, F and G). Adenine-treated mice showed an approximately 80% reduction in capillary VEGF-A expression (*P* = 0.006; Figure 8, A and B). These data taken together, CKD mice showed lower β-catenin expression in muscles and capillaries and capillary density, all of which corroborated with poor perfusion recovery in CKD mice.

Our regression analysis revealed that blood levels of IS in mice inversely correlated with the capillary density measured by the ratio of CD31^+^/SMA^+^ (*R^2^* = 0.40, *P* < 0.001), β-catenin in the capillaries (*R^2^* = 0.78, *P* < 0.001), and VEGF-A expression in the capillaries (*R^2^* = 0.68, *P* < 0.001) (Figure 8, C–E). We correlated AHR and Wnt activity in ECs in response to sera obtained from CKD mice. Our results showed a 2.5-fold downregulation in EC Wnt activity (*P* < 0.001) and an approximately 1.8-fold upregulation of AHR activity (*P* = 0.002) in response to sera from CKD mice (Supplemental Figure 12, H and I). EC AHR activity negatively correlated with Wnt activity (*R^2^* = 0.518, *P* < 0.001; Supplemental Figure 12J). IS levels inversely correlated with EC Wnt activity (*R^2^* = 0.897, *P* < 0.001). AHR activity in ECs induced by the sera of CKD mice correlated negatively with the capillary density in such mice (*R^2^* = 0.520, *P* = 0.018; Figure 8, F and G). A similar inverse correlation was observed in CKD mice’s blood levels of Kyn and their capillary density, normalized β-catenin, and VEGF-A expressions in the capillaries of these mice (Supplemental Figure 13, A–D). The blood levels of Kyn in CKD mice negatively correlated with Wnt activity in ECs (Supplemental Figure 13E). All these results strongly suggested that the circulating levels of IS and Kyn in blood and AHR activity induced by their sera suppressed Wnt signaling in ECs and postischemic angiogenesis in CKD mice.

### AHR inhibitor normalizes postischemic angiogenesis in CKD mice to a non-CKD state.

**Figure 9:**
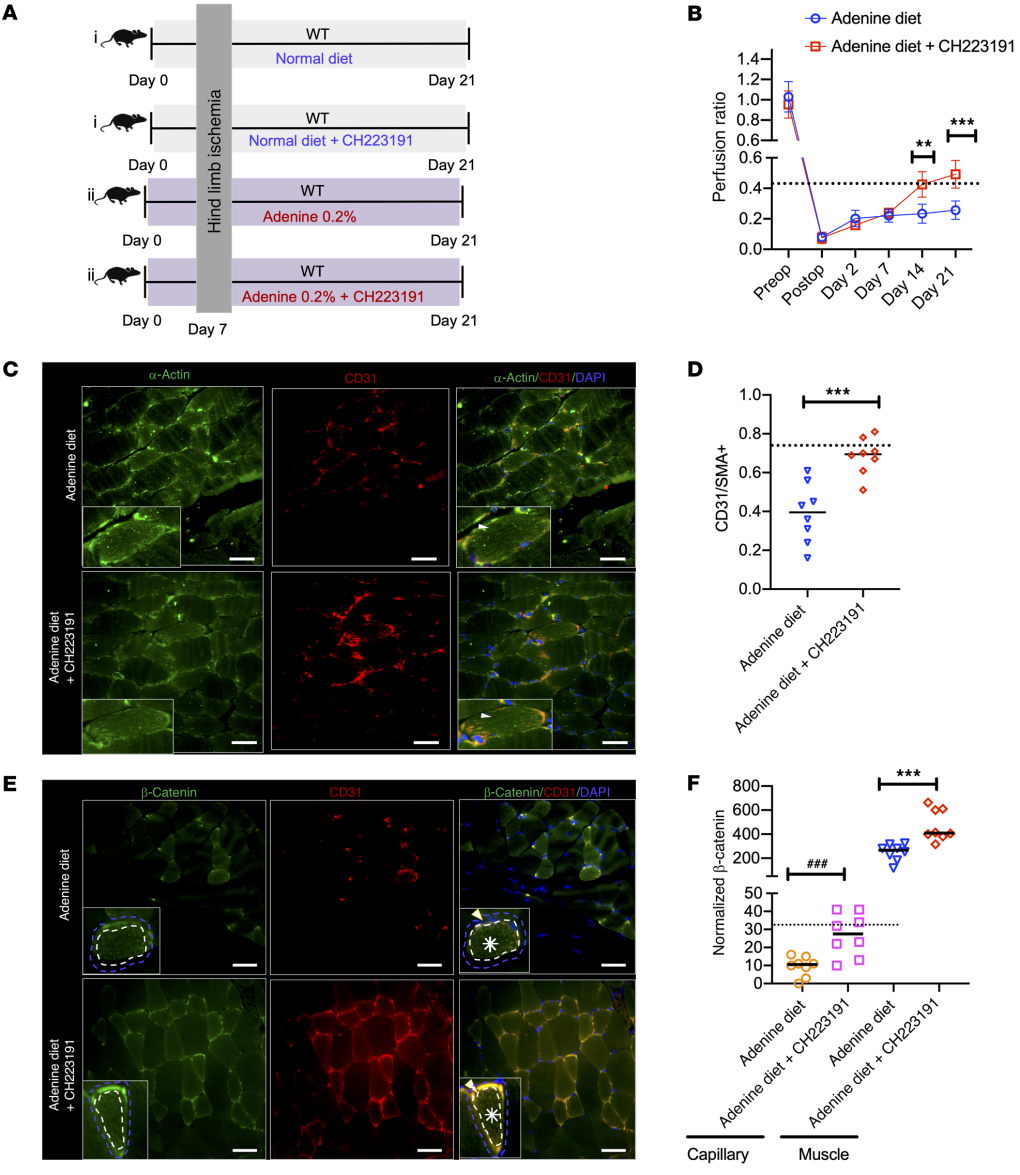
AHR inhibition normalizes postischemic angiogenesis in CKD mice to a non-CKD level. (**A**) A group of C57BL/6 female mice on a 0.2% adenine diet (*n* = 16) or the control diet (*n* = 16) for 7 days underwent HLI. The mice were randomized to 2 groups (*n* = 8/group) and initiated on DMSO or CH223191 for 5 days on and 2 days off for 2 weeks. (**B**) Average perfusion ratios. *n* = 8 mice/group. Error bars show SD. Independent Student’s *t* tests were applied at each time point. The dotted line in this graph and subsequent figures corresponds to the respective values of mice on normal diet. **P = 0.04 for day 14; ***P = 0.001 for day 21. (**C**) Representative stained images from 3 random images per mouse (*n* = 8 mice/group) of the posterior calf muscles from ligated limbs of mice. Insets represent myocytes. White arrowhead is directed at α-actin expression. Scale bar: 25 μm. Original magnification ×400. (**D**) Integrated density of CD31 normalized to α-actin described in Figure 7C. Line represents median value. Student’s *t* test was performed. ***P < 0.001. (**E**) Representative stained images from 3 random images per mouse (*n* = 8 mice/group) of the posterior calf muscles from ligated limbs of mice. Insets show a representative myocyte stained with β-catenin along with surrounding capillaries. Blue dotted lines represent ROI of a myocyte and capillaries; and white dotted lines represent ROI of a myocyte. White asterisks correspond to β-catenin in a myocyte. White arrowheads are directed to β-catenin in the capillary. Scale bars: 25 μm. Original magnification ×400. (**F**) The integrated densities of normalized β-catenin in muscles and capillaries from 8 mice per group. Lines represent median. Student’s *t* test was performed. For capillary ^###^*P* = 0.002 and skeletal muscle ***P = 0.007.

The role of AHR signaling in mediating the above-described effects in CKD mice was examined using AHR inhibitors. A total of 32 mice were initiated on an adenine diet (30, 31) or normal diet (*n* = 16/group) (Figure 9A). After 7 days of the diet, the mice underwent HLI and were randomly divided to receive CH223191 (10 mg/kg, *n* = 8) or vehicle (DMSO, *n* = 8) once a day by i.p. injection for 5 days on and 2 days off for 17 days on adenine or normal diet. Seven days of adenine diet resulted in BUN of 47.3 ± 5.8 mg/dl, corresponding to early stage CKD. This experimental strategy mimics PAD initiation in humans at the early stages of CKD and its progression with deterioration of renal function (4). At the end of the experiment, mice on an adenine diet showed significantly higher BUN. However, there was no difference in BUN in mice with an adenine diet plus CH223191 compared with the vehicle-exposed adenine mice (Supplemental Figure 14A).

Compared with the mice on a normal diet, the mice treated with normal diet plus CH223191 showed an increase in the perfusion ratio at 21 days (*P* = 0.012) and capillary density as measured by normalized CD31^+^SMA^+^ cells (*P* = 0.041). The mice on a normal diet plus CH223191 also demonstrated an upregulation of capillary β-catenin in the capillaries by approximately 30% (*P* = 0.026), VEGF-A by 27% (*P* = 0.053), and skeletal muscle β-catenin by approximately 50% (*P* = 0.006; Supplemental Figure 14, B–E) compared with control mice.

**Figure 10:**
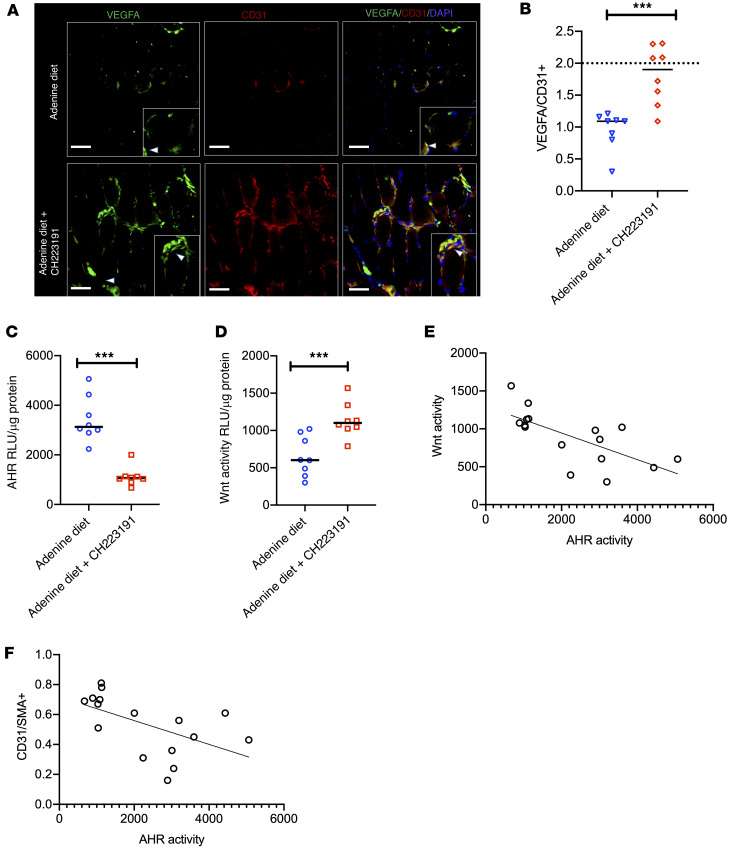
AHR inhibition improves angiogenic response in CKD mice. (**A**) Representative images of the posterior calf muscles from ligated limbs of mice from 3 random images per mouse (*n* = 8 mice/group). Insets show myocytes with white arrowheads directed to VEGF or CD31. Scale bars: 25 μm. Original magnification ×400. (**B**) The integrated density of normalized VEGF-A to CD31 from images in Figure 7G. Line represents median. Student’s *t* test was performed. ***P < 0.001. (**C**) ECs expressing XRE promoter luciferase reporter were treated with 1% sera from adenine-treated mice with or without CH223191. Average for 6 luciferase-independent repeats is shown. Error bars show SEM. Student’s *t* test was performed. AHR activity. ***P < 0.001. (**D**) ECs expressing LS were treated with 1% sera from mice from both groups. Average for 6 independent repeats of Wnt activity is shown. Error bars show SEM. Student’s *t* test was performed. ***P < 0.001. (**E**) Correlation between AHR activity and Wnt activity in ECs in response to sera from adenine-treated mice with or without CH223191. (**F**) A correlation between the EC AHR activity in response to the sera from mice listed in Figure 9A and their capillary density in muscles of ligated limb.

AHR inhibition improved these postischemic angiogenesis parameters to a greater extent in CKD mice. An approximately 50% increase in the perfusion ratio was observed within 7 days of treatment with CH223191, and this pattern continued until the end of the experiment (Figure 9B, compared with mice on adenine diet, 1 vehicle, mice on adenine diet plus CH223191, *P* = 0.04 for day 14 and *P* = 0.001 in day 21). Notably, an increase in the perfusion ratio in CKD mice treated with CH223191 was comparable to that in mice on a normal diet (non-CKD) (shown as a dotted line Figure 7B). Compared with adenine mice treated with vehicle, adenine mice on CH223191 showed a 2-fold increase in capillary density (*P* = 0.004), a 3-fold increase in β-catenin expression in the capillaries (*P* = 0.002), a 2-fold increase in β-catenin expression in skeletal muscles (*P* = 0.007), and an approximately 1.8-fold increase in VEGF-A expression in the capillaries (*P* = 0.006; Figure 9, C–F, and Figure 10, A and B). Importantly, there were no differences in these parameters in CKD mice treated with CH223191 compared with mice on a normal diet (non-CKD; represented as dotted lines in Figure 9, B, D, and F, and Figure 10B). All these data suggested that the AHR inhibitor normalized postischemia angiogenesis in CKD mice to a non-CKD level.

We further examined the correlation between EC AHR and Wnt activities induced by sera from CKD mice with their angiogenic parameters (Figure 10, C–F). Sera of CKD mice exposed to CH223191 showed an approximately 2-fold suppression of AHR activity in ECs compared with sera derived from vehicle-treated adenine mice (Figure 10C). Compared with sera from vehicle-treated adenine mice, EC Wnt activity was 1.8-fold higher in response to sera from mice administered CH223191 (Figure 10D). AHR activity in ECs inversely correlated with Wnt activity in ECs (*R^2^* = 0.48, *P* = 0.002) (Figure 10E). There was a strong and significant inverse correlation between serum-induced AHR activity in ECs and capillary density in the mice from which the serum was derived (*R^2^* = 0.39, *P* = 0.030) (Figure 10F). These studies suggested that CH223191 treatment suppressed AHR activity in ECs and upregulated Wnt activity in ECs to improve the postangiogenic response in muscles of CKD mice.

### Trp metabolite levels correlate with adverse limb outcomes in PAD patients.

To examine the relevance of our findings to humans, we used 2 separate cohorts of patients. A cohort of ESKD patients without PAD was probed with the hypothesis that their sera would induce a higher AHR activity and greater suppression of Wnt activity in ECs and that these activity levels would correlate with the levels of Trp metabolites. ECs expressing LS or an XRE-responsive promoter were exposed to individual serum from CKD patients (uremic sera) and compared with sera derived from age-, sex-, and ethnic background–matched controls (Supplemental Table 1). We had previously reported significantly higher levels of IS in these uremic sera (24). Our expanded analysis showed higher levels of Kyn in them (uremic sera, 351 ± 217 nM vs. controls, 124.1 ± 109.9 nM; *P* < 0.001). Exposure of ECs to uremic sera resulted in greater AHR activity (*P* < 0.001) and suppressed Wnt activity (*P* = 0.017) compared with those treated with control sera. EC Wnt activity inversely correlated with the levels of IS (*R^2^* = 0.34, *P* < 0.001) and Kyn (*R^2^* = 0.25, *P* = 0.025) in ESKD patients (Supplemental Figure 15, A and B).

We further sought to determine the relevance of Trp metabolites in the pathogenesis of PAD complications in a clinical sample. We examined a nested case-control study from a cohort of patients with lower extremity PAD (defined as ankle-brachial index [ABI] < 0.9 a or history of prior revascularization) who were followed prospectively for 2 years for PAD clinical events. Of the entire cohort of 348 patients, 28 patients who experienced an adverse limb event had adequate remaining plasma samples for metabolite measurement. These patients were matched by age, sex, and estimated glomerular filtration rate (eGFR) to 35 PAD patients without adverse limb events (control group). The PAD patients with metabolite measurements were older, but otherwise were like the patients without metabolite measurements. Of the 28 patients with adverse limb events, the events included new or worsening claudication (*n* = 9), CLTI (*n* = 8), limb loss (*n* = 2), graft or stent thrombosis (*n* = 2), any amputation (*n* = 6), stenosis/restenosis (*n* = 7), and new revascularization procedures (*n* = 8). Patients may have had more than one event, but first event date was used for analysis. The events were adjudicated by a panel of 3 physicians using prespecified definitions. Plasma samples collected at baseline were stored at –80°C. There were no differences in the clinical characteristics of PAD patients who experienced a limb event compared with those without limb events (Table 1).

The plasma of PAD patients who subsequently developed adverse limb events showed higher IS levels (5.8 ± 5.3 μM) than that of PAD patients without adverse limb events (3.45 ± 2.9 μM, *P* = 0.028). Similarly, Kyn levels were 2.2-fold higher in PAD patients with adverse limb event (308.5 ± 215.1 nM vs. controls 141.6 ± 45.26 nM, *P* < 0.001), KA (PAD with adverse limb event, 776.1 ± 519.7 nM vs. controls, 521.6 ± 302.5 nM, *P* < 0.001), and XA (PAD with adverse limb event, 3.9 ± 1.9 μM vs. controls, 2.61 ± 0.85 μM, *P* < 0.001). There were no differences in the levels of Trp, AA, and QA between the 2 groups. Notably, the concentration range of IS and Kyn found in PAD patients with events downregulated Wnt/β-catenin activity in ECs to a greater extent (Figures 1 and 2 and Supplemental Figure 8, B–D) compared with those levels seen in PAD patients without event.

The plasma from PAD patients with adverse events activated AHR activity in ECs 60% more compared with the group without adverse events (*P* = 0.017) and suppressed Wnt activity in ECs to a greater extent compared with plasma derived from PAD patients without adverse limb events (*P* = 0.032). Cox’s proportional hazards model was developed for the incidence of adverse limb events adjusting for age, sex, and eGFR. Higher plasma levels of IS, Kyn, KA, XA, and the extent of EC AHR induction and Wnt suppression by these plasma samples were associated with greater risk of adverse limb events (Table 2). In contrast, AA and QA were not associated with adverse limb events. These findings suggested that Trp metabolites with Wnt-suppressive effect in ECs (Supplemental Figure 7) were higher in PAD patients with adverse limb events and contributed to the risk of adverse limb events in them.

## Discussion

While the general mechanisms of PAD pathogenesis are well delineated, there is a distinct paucity of studies elucidating the risk-specific mediators of PAD. Addressing this knowledge gap in CKD, we demonstrate that a specific set of uremic solutes derived from Trp metabolism downregulates Wnt signaling through the AHR pathway in ECs and postischemic angiogenesis and induces capillary rarefaction in CKD mice. These specific Trp metabolites substantially increase the risk of future adverse limb events in PAD patients. This work uncovers a mechanistic link between Trp-derived uremic solutes with PAD and AHR as a potential therapeutic target in PAD in CKD patients.

### Trp metabolites and PAD.

Several Trp metabolites induced a similar cellular effect (Wnt/β-catenin suppression) in different cell types. These observations along with other examples (12, 13, 23, 24, 30) support a “group” effect of a set of uremic toxins. This phenomenon can be explained by the mediation of their effect through AHR and creates an opportunity to target AHR to suppress the effects of several toxins simultaneously (34). Endothelial dysfunction is universal in CKD patients and is known to be mediated in part by uremic solutes such as IS (11). The results of the current work raise the possibility of involvement of Wnt signaling in EC dysfunction in CKD patients. β-Catenin plays important roles in skeletal muscle energy metabolism, mitochondrial health, and structural integrity (35). Downregulation of β-catenin in CKD mouse muscles may explain the sarcopenia in CKD patients (36). There are cell type–specific E3 ligases of β-catenin targeting its specific regions. IS regulating β-catenin through its degron motif points to a specific E3 ligase, which warrants further investigation.

### The translational potential of the Trp metabolite/Wnt/β-catenin angiogenesis axis.

These data suggest that IS and Kyn increase the risk of PAD initiation and progression up to complications with renal deterioration, a notion supported in clinical studies (37). Not all CKD patients are at an equal risk for PAD or its complications (6). The current study strongly suggests that CKD stage alone based on creatinine is insufficient for stratifying PAD patients and supports studies exploring the biomarker potential of these uremic solutes to stratify CKD patients at risk for PAD and its complications. Interestingly, the AHR inhibitor in CKD models normalized the postischemic angiogenesis to a non-CKD level in a manner similar to that in uremic thrombosis (30). AHR inhibitors are undergoing clinical trials for other indications (38) and can be repurposed for PAD in CKD.

The adenine mouse model was selected for high IS and Kyn levels and its ability to recapitulate uremic features. However, this model is not characterized by proteinuria, a known risk factor of PAD (4). Postischemic angiogenesis is a complex process dynamically orchestrated by several cell types, including immune cells, pericytes, and vascular smooth muscle cells, etc. Further work is needed to generate a detailed mechanistic understanding of the effects of uremic toxins on different cell types involved in angiogenesis and skeletal muscles in the context of PAD.

This work provides what we believe is the first mechanistic link among a group of uremic toxins, Wnt signaling, and PAD and paves the way for delineating risk-specific mediators of PAD. This study serves as a foundation for future investigation to examine the predictive power of Trp metabolites for refining the stratification of CKD patients at risk for PAD and explores AHR inhibitors as a therapeutic target for PAD complications.

## Methods

### Human subjects.

Human subjects, laser doppler analysis, IHC, the HLI model, the adenine CKD model, the IS-specific solute model, cell culture and chemical treatment, generation of the AHR CRISPR cell line, subcellular fractionation, Wnt activity, immunoblotting, 3[H] thymidine incorporation assay, quantitative reverse-transcriptase PCR (qRT-PCR), and ELISAs and zebrafish angiogenesis assays are described in the Supplemental Methods.

### Statistics.

Statistical analysis was performed using GraphPad Prism 8 (GraphPad Software) or SAS, version 9.3 (SAS Institute). Results are expressed as the mean ± SEM or median, range, and 25th and 75th percentiles in box-and-whisker plots. Two-tailed Student’s *t* test was performed for group comparisons. For multiple groups, overall group comparison was first examined using ANOVA. With the rejection of the null hypothesis, post hoc analysis using Bonferroni’s correction or the pairwise comparisons with Tukey’s multiple comparison procedure was performed for in vitro and in vivo studies. Linear regression was performed to correlate 2 parameters. Cox’s proportional hazard model was developed after adjusting for confounders for human study. *P* ≤ 0.05 was considered statistically significant.

### Study approval.

Both the human and animal studies were conducted with the approval of the IRB at Boston University (H-26367) and the IACUC (PROTO201800298) and Institutional Biosafety Committee (IBC). Human subjects provided written, informed consent.

## Author contributions

NVA is the first author in the list of shared first authors as she initiated the project, developed the animal models of PAD and completed key experiments. WY performed all the mechanistic cell-based studies and completed remaining experiments to bring this manuscript to completion. VCC conceived of the hypothesis. VCC, NR, NVA, and WY designed the research. NVA, MB, and SL performed the HLI. WY, MAN, SR, SL, and JAW performed the experiments. VCC, MAN, and MB performed IF studies. JAW, MHK, DS, JDR, JJS, SA, AF, and JF provided scientific input and data analyses. SAW and NL performed the metabolomics analysis and provided their interpretation. AF and NMH performed the prospective human study. NMH performed the analysis of the BMC PAD cohort. VCC performed the analysis of in vitro and in vivo studies. VCC, NVA, and WY prepared the manuscript. All authors edited the manuscript.

## Supplementary Material

Supplemental data

## Figures and Tables

**Table 2:**
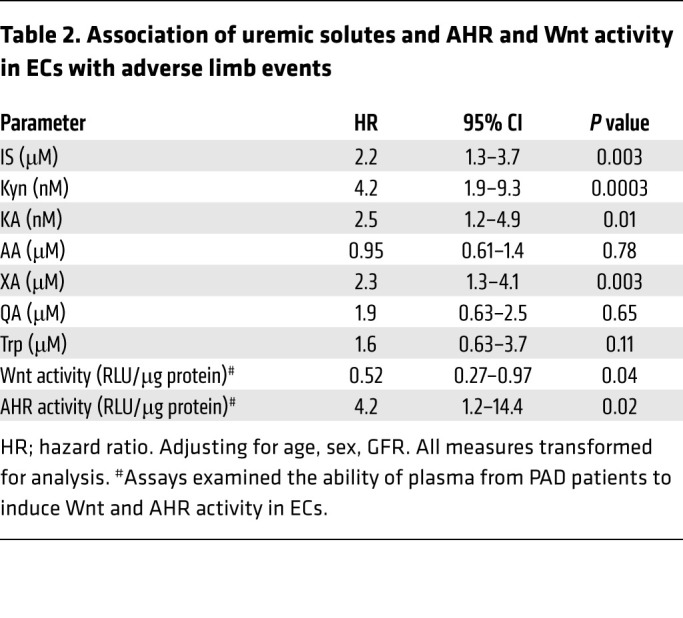
Association of uremic solutes and AHR and Wnt activity in ECs with adverse limb events

**Table 1:**
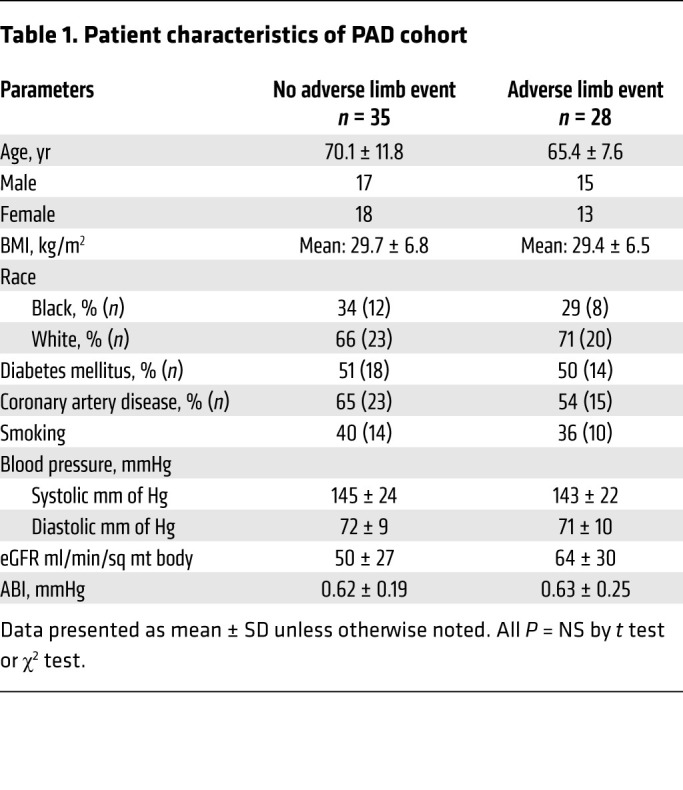
Patient characteristics of PAD cohort

## Version 1 11/09/2021

In-Press Preview

## Version 2 01/04/2022

Electronic publication
