# Supplementary material for: Tryptophan metabolites suppress the Wnt pathway and promote adverse limb events in chronic kidney disease
Source: J Clin Invest. 2022 Jan 4;132(1):e142260. doi: 10.1172/JCI142260 (PMC8718145; doi:10.1172/JCI142260)

Supplemental information

Material and Methods

Human subjects

Two cohorts of human subjects were used in this study. The first group consisted of participants with CKD on hemodialysis. The pre-dialysis sera samples from them (uremic sera, N =20) and their age, sex and ethnic background-matched controls (control sera, N = 15) were collected, as described previously[24, 25]. Both pooled and individual sera were used for our experiments. CKD patients with hemoglobin <8 gm/dl and non-CKD controls with creatinine more than 1.0 mg/dl were excluded (Supplemental Table 1). The second group consisted of patients with PAD (defined as ABI <0.9) followed for 2 years. Their baseline sera samples were stored at -80^{0}C.The baseline characteristics of subjects are in Table 1.

Adenine-induced CKD model

Mice were fed a normal chow diet (Teklad Global 18% Protein Rodent Diet, Envigo) supplemented with 0.2% adenine (vitamin B4; diet formulated by Research Diets) as described previously (1, 2). Blood urea nitrogen (BUN) in these animals was determined using the QuantiChrom Urea Assay Kit (DIUR-100) (BioAssay Systems). CH223191 was obtained from Sigma (# C8123) and dissolved in dimethyl sulfoxide (DMSO).

Indoxyl sulfate (IS)-specific solute model

C57BL/6 mice (10-14 weeks old) were given IS ad libitum in water, and the excretion of IS was inhibited by probenecid, an OAT1 and OAT3 channel inhibitor. Mice were exposed to IS dissolved in drinking water at 4 mg/ml, and probenecid was administered at 15 mg/kg by intraperitoneal injection twice daily.

Liquid chromatography–mass spectrometry (LC/MS)

LC/MS for determination of IS was performed using API 400 triple quadrupole mass spectrometry as described previously (3, 4).

Hindlimb Ischemia (HLI) model and harvest of skeletal muscles

All animal surgeries were performed in accordance with our approved Institutional Animal Care and Use Committee protocols at Boston University. A group of 8- to 12-week-old female C57BL/6 wild-type (WT) mice (Jackson Laboratories) underwent unilateral high femoral artery and vein ligation. Mice were anesthetized using continuous isoflurane anesthesia. Using aseptic technique, the left femoral artery and vein were exposed above the level of the profunda and epigastric arterial branches and ligated with 6-0 silk suture. Buprenorphine was used for analgesia.

Mice were euthanized. The chest was opened through a midline sternotomy. A 27G needle was introduced into the left ventricle to perfuse phosphate buffered saline (PBS). The right atrium was incised to exsanguinate the animal and to drain the excess perfusate. For image quantification, the slide in its entirety was scanned by a motorized stage system using the Nikon NIS Elements software at the Molecular imaging core facility at the Boston University School of Medicine. Using ImageJ (5), the signal was first converted to gray scale and thresholding was performed equally for all the images. The number and intensity of pixels were analyzed as integrated density. The area of skeletal muscle was defined as the region of interest (ROI) and measured using ImageJ. The integrated density of all the images were normalized to its area.

Laser doppler blood flow analysis

Scanning laser-doppler perfusion imaging (Moor Instruments, Wilmington, DE) was used to evaluate hindlimb perfusion before ligation, immediately after the procedure, and postoperatively as described previously (6). Mice were placed on sternal recumbency on a heating pad at 37°C to minimize temperature variation. Mean perfusion was recorded from the plantar surface of the hind paws. Perfusion ratio was defined as the ratio of the mean perfusion of the ligated limb to the non-ligated limb.

Histological preparation

At the time of sacrifice, kidneys were harvested from animals and formalin fixed, then fixed in xylene, and paraffin embedded. Tissues were cut into 5 micrometer sections and stained with hematoxylin and eosin for histology.

Protein lysis and immunoblotting and antibodies

Posterior calf muscles (soleus and gastrocnemius) were removed at the time of sacrifice after perfusion of PBS and snap frozen in liquid nitrogen. Snap frozen posterior calf muscles were subjected to protein extraction using RIPA buffer (Boston Bioproduct, MA). Tissue was then homogenized using a sonicator and bead homogenizer (Company, City, State). Protein lysates were used for western blotting (WB) and tissues for immunofluorescence (IF), as indicated in the table below.

Antibody	Vendor	Catalogue	Dilution (WB/IF)
β-catenin	Cell Signaling	#8480	1:1000/1:100
Active β-catenin	Cell Signaling	#19807	1:1000
Myc-tag	Cell Signaling	#2276	1:500
β-Actin	Santa Cruz	sc-47778	1:1000
GAPDH	Cell Signaling	#5174	1:2500
α-Actin	Abcam	sc-5694	1:1000
Ubiquitin	Cell Signaling	#3933	1:1000
VEGF-A	Abcam	Ab1316	1:100

Cell culture

Primary human dermal microvascular endothelial cells (ECs) (Promocell, Germany) pooled from 3 donors were grown in endothelial growth medium-2 (EGM-2) (Promocell, Germany). EGM-2 was prepared by supplementing endothelial basal medium (EBM-2) with fetal bovine serum (2%), hydrocortisone (1μg/ml), fibroblast growth factor-1 (10 ng/ml), epidermal growth factor (5 ng/ml), insulin-like growth factor (20 ng/ml), ascorbic acid (1 μg/ml) and heparin (90 μg/ml). These primary human cells were tested for Mycoplasma, HBV, HCV and HIV-1 using ELISA. The cells were characterized by staining with anti CD31 and vWF antibodies, by Dil-Ac-LDL uptake, and by smooth muscle actin antibody using immunofluorescence. The cells were used within 2 months of purchase over 8 passages. Primary human umbilical venin endothelial cells purchased from Lonza and grown per the manufacturer's protocol. HK-2 and NIH3T3 cell lines were grown in DMEM 10% FBS + 1% PS and L-glutamine. Primary human skeletal muscle cells were obtained from the lab of Prof Vincent Mouly, Paris and described in detail in the reference (7). The primary human skeletal muscle cells derived from a quadriceps of a 38-year-old male (AB1079) and were grown as recommended in the reference (7).

AHR CRISPR cell line generation

Primary human umbilical vein endothelial cells (Lonza) were used to silence AHR. First, we generated lentiviral particles in HEK 293 cells expressing AHR CRISPR using a single vector system with packaging signal, Rev response element, central polypurine tract, 2A self-cleaving peptide (8) (Addgene, catalogue #52961). The ECs were transduced with these viral particles and the cells were selected with puromycin. The viral particles without AHR CRISPR transgene served as controls.

Ubiquitination and half-life studies

Cells treated with MG132 (Calbiochem) were harvested and immunoprecipitation was performed. The eluents were probed for anti-ubiquitin antibodies, as done previously (1, 9). For half-life studies, the cells were treated with Cycloheximide (CalBiochem) 20 ug/ml prior to the harvest.

Antibodies and reagents

Primary antibodies to the following antigens were obtained as follows: CD31 (Abcam, Cambridge, MA); total β-catenin (BD Biosciences) and active β-catenin (Cell Signaling, Danvers, MA), Alexa Fluor 568-conjugated IgG (Molecular Probes). Anti-mouse actin, tubulin and fibrillarin and VE-cadherin were obtained from Cell Signaling, Danvers, MA. Wnt3a was obtained from R & D systems and was dissolved in PBS + 0.1% BSA, as described previously (10).

Cell transfections and chemical treatment

Cultured cells plated overnight were transiently transfected using Lipofectamine 2000 (Invitrogen) per manufacturer instructions. The cells seeded in 6-well plates were stably transfected with LS and FuLS plasmids as described previously-(11-13). AHR activity was measured using ECs stably expressing Xenobiotic-responsive element (XRE) tethered luciferase described previously (4). After 48 h of transfection, luciferase assays were performed using the Dual Luciferase kit® (Promega) and normalized using protein content determined by Bradford Assay (BioRad). Uremic solutes were obtained as follows and were used at the concentrations observed in patients with CKD. The following uremic solutes were used: Urea 1200 µg/mL (Sigma, U5378), Creatinine 60 µg /mL (Fisher Scientific, C-524), Oxalic acid 5 µg/mL (Sigma-Aldrich, 75688-50G), Indoxyl sulfate 10µM, 50 µM, 100 µM (Arum Pharmaceutical, U26977). Lithium Chloride was obtained from Sigma Aldrich. CH223191 was obtained from Sigma (Catalogue # C8123), as previously described (14, 15).

All other uremic solutes (IS, IA, Kyn, KA, XA, QA and AA) were dissolved in DMSO to generate a stock solution and was added to the full growth media of the ECs to achieve the final concentration as indicated for different experiments. IS and Kyn are albumin-bound uremic solutes. The albumin concentration in the medium was raised by adding human serum albumin to the media to increase the final concentration of protein in the media to 3.5 mg/dL, which corresponds to the average albumin concentration observed in patients with CKD and as performed previously (1, 14) and recommended by EuroTox group (16).

MTT assay and 3[H]Thymidine incorporation assays

Early passage primary human dermal microvascular ECs were seeded in 96 well plate at a density of 10,000/well. Upon treatment with control or uremic sera, the cells were exposed to Alamar Blue® for 24 hours. The absorbance was read at 570nm using Tecan M1000 Pro plate reader.

For the cell proliferation assay, early passage primary human ECs were seeded in 96 well plate at a density of 10,000/well. Cells were serum starved overnight using EGM-2 diluted with EBM-2 (endothelial cell basal medium-2) in 1:5 ratio. The cells were treated with 5% control or uremic serum in EBM-2 for 24 hours, after which they were supplemented with 1μCi of ^{3}[H] Thymidine (Perkin Elmer) overnight. The lysed cells were counted for radioactivity using the LabLogic 300SL Liquid Scintillation Counter.

Wnt signaling in endothelial cells

TCF/β-Catenin-responsive Luciferase Reporter Assay was used for Wnt/β-catenin signaling pathway activity as previously described (10, 11). Briefly, the above-described ECs stably expressing LS or FuLS constructs were firstly starved with serum free medium for 24 hours, then were treated with specific chemicals in 10% FBS culture medium or 5% of sera from controls and

CKD patients for another 24 hours. The total protein level for each condition was measured by the Bradford assay (Bio-Rad). Luciferase assays were performed using the Dual-Luciferase kit (Promega) and normalized with protein levels. Chemicals used in this application included human recombinant Wnt3a (R&D Systems) dissolved in PBS + 0.1% bovine serum albumin, and LiCl (Sigma Aldrich), which were respectively dissolved in autoclaved ddH2O and DMSO.

AHR activity

Activity was measured in ECs as described previously (4). Briefly, the cell lines stably expressing XRE-response element tethered to luciferase reporter (XRE-luc) (Qiagen, CLS-9045L-8) were used. The cells seeded at 1000/well in 96-well plate were serum starved for 16 hours and then treated with the serum with or without AHR inhibitor, CH223191 for 24 hours. Firefly luciferase activity was measured using luciferase assay kit (Promega# E1501). After luciferase assay, the cells were lyzed in RIPA buffer and the protein content was determined using Bradford assay. The luciferase signal was then normalized to protein content (relative luciferase activity unit).

IL-8 and VEGF-A ELISA

Cell culture media were harvested and concentrated using Amicon filters 3Kd (Millipore) and subjected to ELISAs for IL-8 and VEGF-A (R&D systems) per manufacturer's instructions.

qRT-PCR

ECs cells were harvested using Trizole (Sigma). RNA was precipitated using ethanol and purified with a Qiagen column and underwent reverse transcription to cDNA followed by qPCR using

AXIN2 Taqman primers (catalogue number: 4331182) and MYC Taqman primers (Catalog number: 4414113).

Subcellular fractionation

Cells were Dounce homogenized (Kontes) in 250 mM sucrose, 10 mM HEPES, pH 7.4, 2.5 mM $MgCl_2$, 0.5 mM EDTA, 100 μM Na_3VO_4, 100 μM phenylmethylsulfonyl fluoride (PMSF) and complete protease inhibitor mixture (Roche). After two centrifugations at 1,000 x *g* for 10 min at 4 °C, the anucleated supernatant was centrifuged at 100,000 x *g* at 4 °C for 30 min. The supernatant was removed. The nuclear pellet from the first spin was resuspended in 50 mM HEPES, pH 7.4, 50 mM KCl, 300 mM NaCl, 0.1 mM EDTA, 100 μM Na_3VO_4, 100 μM PMSF and complete protease inhibitor mixture, and lysed by three cycles of freeze and thaw. The nuclear fraction was cleared by centrifugation.

Zebrafish angiogenesis assay

Experiments using zebrafish as a model system were approved by the IACUC Animal Welfare Committee (AN-201800388). Fli1-eGFP transgenic adult zebrafish (Danio rerio) were housed in the fish facility at Boston University Medical Campus, as described previously(13, 17). Fertilized eggs were raised in E3 medium (5 mM NaCl, 0.17 mM KCl, 0.33 mM CaCl2, 0.33 mM MgSO4, 1% methylene blue). De-chorionated embryos at 24 hours post-fertilization (hpf) were exposed to IS or Dimethyl sulfoxide (DMSO) for 48 hours. Zebrafish embryos were anesthetized by 0.006% tricaine (Sigma-Aldrich) and photographed under a Nikon fluorescence microscope. The images obtained from randomly selected 10 embryos per group at the same setting of microscope were analyzed.

Statistical Analysis

Statistical analysis was performed using GraphPad Prism 8 (GraphPad Software, La Jolla, CA) or SAS version 9.3 SAS Institute, Cary, NC). Results are expressed as the mean ± SEM or median, range, and 25th and 75th percentile in box and whisker plots. Student's t test was performed for group comparison. For multiple groups, overall group comparison was first examined using analysis of variance (ANOVA). With the rejection of null hypothesis, the post-hoc analysis using Bonferroni correction or the pairwise comparisons with Tukey's multiple comparison procedure were performed for in vitro and in vivo studies. Linear regression was performed to correlate two parameters. A Cox proportional hazard model was developed after adjusting for confounders for human study. P values of ≤ 0.05 were considered statistically significant.

Table S1. Patient characteristics

Parameters	Control group<br>N= 15	End stage renal disease group<br>N = 20
**Age (**years)	33 ± 27	42 ± 15
**Sex** (male, %)	60%	52%
**Race**		
African-American	60%	71%
Caucasian	20%	15%
Hispanics	6%	8%
Others$	14%	6%
**Comorbidity**		
DM*	42%	61%
HTN*	12%	90%
Cardiovascular disease*	0%	32%
Cerebrovascular disease#	0%	20%
**Cause of CKD**		
DM	0%	55%
HTN	0%	30%
Glomerulonephritis	0%	10%
Others[#]	0%	5%
**Medications**		
Aspirin	0%	42%
Plavix	0%	10%
Warfarin	0%	18%
Heparin, Rivaroxaban, Apixaban, Dabigatran	0%	10%

*indicates $p < 0.05$ using Fischer's exact test.
*Cardiovascular disease consists of coronary artery disease, atrial fibrillation, and heart failure (preserved and reduced EF).
#Cerebrovascular disease consists of strokes and intracranial hemorrhages

$ Others includes polycystic kidney disease, recurrent acute renal failure, cardio-renal syndrome, obstructive uropathy, congenital abnormalities of kidney and urinary tract, etc.

Table S2. Circulating levels of a set of uremic solutes in patients at different stages of CKD# (4, 14, 18, 19)

**CKD stage**<br><br>**eGFR ml/min/1.73 sq mt**	**IS (µM)**	**Kyn (µM)**	**KA (µM)**	**QA (µM)**
**Stage 1 eGFR >90**	7.04-14.07	1.71-2.52	0.20-0.53	1.24-1.98
**Stage 2 eGFR 60-89**	0.47-37.52	2.17-2.96	0.21-0.46	1.37-1.95
**Stage 3 eGFR 30-59**	11.73-46.90	2.68-4.12	0.25-0.73	1.47-2.35
**Stage 4 eGFR 15-29**	14.07-93.80	2.31-4.32	0.20-0.88	1.66-3.23
**Stage 5 eGFR <15**	4.69-187.61	4.87-6.41	3.09-6.01	5.38-7.37

NKF guideline: Stage 1 with normal or high GFR (GFR > 90 mL/min), Stage 2 Mild CKD (GFR = 60-89 mL/min), Stage 3A Moderate CKD (GFR = 45-59 mL/min), Stage 3B Moderate CKD (GFR = 30-44 mL/min), Stage 4 Severe CKD (GFR = 15-29 mL/min), Stage 5 End Stage CKD (GFR <15 mL/min)

Total number of subjects in different studies were – N= 877 reference # 4, N= 20 reference number 14, N=139 reference # 18, N = 60 reference number 19.

Supplemental Figure legend

Supplemental figure 1

(A). The ECs were treated with pooled control and uremic serum at the final concentration of 5% in the media for 24 hours and subjected to MTT cell viability assay. Uremic sera were obtained from 20 CKD patients on hemodialysis and their age, sex and ethnic background matched subjects served as controls. Table S1 contains their baseline characteristics. Color change was monitored using a multi-well plate reader. Box plots from six independent samples are shown and compared using a Student's t-test. For this and subsequent figures, the line in the box represents the median, the box spans from the 25th to 75th percentile and the whiskers correspond to the maximal and minimal values. Student's t-test was applied. **$p < 0.01$.

(B). ECs were serum-starved in 1/5th EGM-2 medium then stimulated with 50 ng/ml of Wnt3a to stimulate proliferation along with 5% pooled control or uremic serum for 24 hours. The cells were treated with 1 µCi of ^{3}[H] Thymidine for 16 hours. The lysates were subjected to a scintillation counter. Student's t-test was applied. $p < 0.001$.

(C). Confluent monolayers of primary human endothelial cells were treated with 5% pooled control or uremic serum for 24 hours followed by a scratch assay. Cells were imaged at a regular interval using Nikon Deconvolution Epifluorescence Microscope Representative image from five independent experiment are shown. The dotted black line represents the scratch in both the samples.

(**D**). Digitized images from the above assay were annotated in ImageJ. The migration of cells from the leading edge was calculated from time 0 to time 36 hours. The distance of the leading edge of the wound is represented for the both the groups as box plot. Student's t-test was applied. **$p = 0.0169$.

(E). Primary human dermal microvascular endothelial cells were treated with 5% control and uremic serum. Densitometry analysis of total and active β-catenin normalized with Actin was performed. Average of three independent experiments are shown. Scale bar= SEM. Independent Student's t-tests were performed. Compared to control serum, total β-catenin active β-catenin with uremic serum *** and ### $p < 0.001$

(F). ECs expressing the β-catenin-responsive promoter tethered to luciferase reporter construct (LS) and β-catenin-unresponsive promoter-reporter construct (FuLS) were treated with 5% pooled control or uremic sera. The luciferase signal was normalized to the protein (relative luciferase unit). Average of three experiments performed in triplicate is shown. Error bars = SEM. Student's t-test was applied. ** $p = 0.019$.

(G). ECs pre-treated with 5% control or uremic sera for 24 hours were washed and grown in EGM-2 medium containing 0.1% FBS for 24 more hours. IL-8 ELISA was performed in the harvested medium and the cells were counted. IL-8 levels were normalized to the number of cells and shown as box plots from four independent experiments and each sample done in triplicate. The line represents media, the box represents 25th and 75th percentile and whiskers correspond to the maximum and minimal values. Student's t-test was performed. ** $p = 0.004$.

(H). On the same media, ELISA for VEGF-A was performed and the levels were normalized to the number of cells and shown as box plots from four independent experiments and each sample done in triplicate. Student's t-test was performed. ** $p < 0.001$.

(I). Serum-starved ECs were treated with control or uremic serum for 12 hours and probed by western blotting for Cyclin D1 and actin. Representative images from four independent experiments are shown.

(**J**). Cyclin D1 expression was normalized to actin from four independent experiments and presented as a box-whisker plot. Student's t-test was performed. ** $p = 0.005$.

(K) ECs treated with pooled control and uremic sera and were subjected to qRT-PCR for the Wnt target genes. GAPDH served as a loading control. The cT values were normalized to control. Average of two independent experiment done in triplicates is shown. Error bars = SD. Independent Student's t-tests were performed to compare the groups. ** $p = 0.010$, *** $p = 0.003$.

(**L**) Early passage primary microvascular ECs were treated with 5% control and uremic serum. In parallel, the cells were treated with heat-inactivated serum. The cell harvested at 16 hours of treatment underwent subcellular fractionation. The nuclear fraction was probed for β-catenin antibody and fibrillarin served as a marker of nuclear fraction and loading control. Representative images from four independent experiments are shown.

(M) Densitometric analysis was performed using ImageJ on β-catenin bands which were normalized with fibrillarin as loading control. Average of four experiments are shown. Error bars = SD. Independent Student's t-tests were performed. ** Compares control serum with heat inactivated control serum ** $p = 0.01$. Compared to uremic serum, heat inactivated uremic serum was *** $p = 0.006$. NS= non-significant

There was no difference in the β-catenin levels in ECs treated with uremic serum and heat-inactivated uremic serum (depicted as ns).

(N). Densitometry analysis was performed on endothelial cells treated with the water-soluble uremic toxins. β-catenin was normalized with Actin. Average of three independent experiments are shown. Scale bar= SEM.

Supplemental figure 2

(**A**). NIH3T3 cells treated with a range of concentration of IS underwent subcellular fractionation. The cytosol and nuclear fractions were probed for β-catenin. Tubulin and Fibrillarin were used the markers of subcellular fractions and the loading control. Representative images from three independent experiments are shown.

(**B**). Densitometry was performed on β-catenin in different fractions and normalized to the loading controls for their respective fractions. Average of three independent experiments is shown. Error bars = SD. Student's t-test with Bonferroni's correction was performed. Compared to IS = 0 μM, * $p = 0.012$ and # $p = 0.009$

(**C**). HK2 cells were treated with a range of concentration of IS and then fractionated as above. Representative images from three independent experiments are shown.

(**D**). Densitometry was performed on β-catenin in different fractions as above. Average of three independent experiments is shown. Error bars = SD. Student's t-test with Bonferroni's correction was performed. Compared to IS = 0 μM, * $p = 0.01$.

(E). Primary human skeletal muscle cells obtained from a healthy 38-year-old male were treated with IS concentrations corresponding to the patients with different CKD stages. The lysates were probed for β-catenin. Actin served as a loading control. DMSO treated cells served as controls. Representative images of three independent experiments are shown.

(F). Densitometry analysis of β-catenin normalized with Actin in cells treated with human sera was performed. Average of three independent experiments are shown. Scale bar= SEM. Student's t-test with Bonferroni's correction was performed. Compared to DMSO-treated cells, IS 5 μM $p = 0.007$, IS 10 μM $p = 0.004$, IS 50 and 100 μM $p <0.001$.

Supplemental figure 3

(A). ECs were treated with the serum from subjects with normal renal function (control serum) spiked with the IS at the indicated concentrations. The cells were placed in the hypoxia chamber for 16 hours. The nuclear fractions were probed with fibrillarin as a marker and the loading control. Cells grown at 21% O2 saturation served as controls (Normoxia). Representative images from four independent experiments are shown

(B). Densitometry was performed on all the blots were performed using ImageJ and the β-catenin band was normalized to fibrillarin. Averages for four independent experiments performed under normoxia and hypoxia are shown. Error bars = SD. ANOVA test was performed to compare all the groups $p < 0.001$. Independent Student's t-tests were performed at individual IS concentrations. Compared to normoxia, hypoxia induced greater downregulation of nuclear β-catenin with IS at 1, 5 and 10 μM. * p IS = 1 μM ** p = 0.004, IS = 5 μM * p = 0.011, IS 10 μM * p = 0.010. No significant difference in the nuclear β-catenin was noted between normoxia and hypoxia at IS 50 and 100 μM concentrations.

(C). ECs stably expressing β-catenin-responsive promoter and luciferase reporter (LS construct) were treated with IS at the indicated concentration in the normoxia and in the hypoxia chamber. The luciferase assay was performed and normalized to the protein content. Averages for six independent repeats are shown. Error bars = SD. ANOVA test was performed to compare all the groups $p < 0.001$. Independent Student's t-tests were performed at individual IS concentrations. Compared to normoxia, at IS at 0, 1, 5 and 10 μM, hypoxia induced greater downregulation of nuclear β-catenin. ** p IS 0 μM = 0.010, IS = 1 μM * $p < 0.001$, and IS = 5 μM # $p < 0.001$, IS = 10 μM ## p = 0.004.

Supplemental figure 4

(A). ECs transfected with different β-catenin plasmids were lyzed and probed with anti-Myc antibodies. The DNA used for transfection was adjusted to obtain comparable expression between them. Actin served as a loading control. Representative blots from three experiments are shown.

(B). Densitometry analysis of β-catenin constructs normalized to actin from the above experiments are shown. Average of three independent experiments is shown. Error bar = SD. Independent Student's t-tests were performed. Compared to vehicle treated cells, IS-treated samples for β-catenin wild type * $p = 0.003$, and β-catenin delC * $p = 0.05$ and # $p = 0.001$.

(**C**). ECs were transfected with Myc-tagged wild-type and S33A β-catenin were serum staved and exposed to IS (10 μM) or DMSO for 24 hours. The cells were fixed and stained with Myc antibody and secondary staining with Alex fluro® 488. DAPI was used to stain nuclei. Representative images are shown. Myc-tagged S33A β-catenin expressed in the nuclei.

(**D**). ECs were transfected with Myc-tagged wild-type and S33A β-catenin and then treated with IS 10 μM. The cells were treated with Cycloheximide (30 μg/ml) for indicated time and cells were harvested and probed for Myc tag. Actin served as loading controls. Representative images from four independent experiments are shown.

Supplemental figure 5

(**A**). Densitometry quantitation of polyubiquitinated wild-type and S33A β-catenin in ECs exposed IS in Figure 3F. The ubiquitinated β-catenin band was normalized to immunoprecipitated β-catenin. Average of three experiments are shown. Error bars = SD. Student's t-test was applied. **$p = 0.015$

(**B**) ECs pre-transfected with the Myc-tagged β-catenin wild-type or Myc-tagged β-catenin S33A and DMSO or IS for 24 hours. The cells were washed and grown for 24 more hours. IL-8 ELISA

was performed in the harvested medium and normalized to the number of cells. Average of three independent experiments done in duplicate is shown. Error bars = SD. Independent Student's t-tests were performed. Compared to the control plasmid (control depicted as light blue bars) # $p = 0.001$ for wild-type β-catenin and $p < 0.001$ β-catenin S33A. Compared to β-catenin wild-type treated with DMSO, IS treatment suppressed IL-8. * $p = 0.03$ and ** $p = 0.004$.

(**C**). The medium from the above was experiment was examined for VEGF ELISA as above. Average of three independent experiments done in duplicate is shown. Error bars = SD. Independent Student's t-tests were performed. Compared to the control plasmid (control depicted as light blue bars) # $p = 0.004$ for β-catenin wild-type and $p < 0.001$ β-catenin S33A. Compared to β-catenin wild-type treated with DMSO, IS treatment suppressed Wnt signaling * $p = 0.01$ and ** $p = 0.009$.

(**D**). ECs pre-transfected with the Flag-tagged wild-type and increasing concentration of Myc-tagged β-catenin S33A and DMSO or IS for 24 hours. The total amount of DNA was maintained same in each condition. The cells were washed and grown for 24 more hours. IL-8 and VEGF-A ELISAs were performed in the harvested medium and normalized to the number of cells. Average of three independent experiments done in duplicate is shown. IL-8 and VEGF-A are presented as ng/ml/10^4 cells and pg/ml/10^4 cells. Error bars = SD. Independent Student's t-tests were performed. IL-8: Compared to β-catenin wild-type treated with DMSO # $p = 0.001$. Compared to β-catenin wild-type treated with IS ** $p = 0.01$. Similarly, VEGF-A group: # $p = 0.002$ and ** $p = 0.02$.

(**E**). Lysates of ECs transfected with Flag-tagged β-catenin wild-type and Myc-tagged β-catenin S33A treated with DMOS or IS were probed in two separate gels for the expression of plasmids.

Representative images of experiments quoted in Figure 3H and Supplemental Figure 5D are shown.

Supplemental figure 6

Dietary Trp is processed by gut microbiome to generate indole, which is subsequently converted to IS and IA in liver. Majority of dietary Trp is absorbed from gut and is converted to Kyn in liver. Kyn subsequently undergoes several steps of processing to anthranilic acid (**AA)**, Kynurenic acid (**KA**), Xanthurenic acid (**XA**) and Quinolinic acid (**QA**),

Supplemental figure 7

(**A**) ECs stably expressing LS construct were treated with increasing concentration of lithium chloride. The cells were lyzed and luciferase was measured as relative luciferase activity and normalized to the protein content (RLU). Averages for six independent repeats are shown. Error bars = SD. ANOVA test was performed to compare all the groups. $p < 0.001$ for lithium chloride. (**B-H**) Wnt activity was measured in ECs expressing LS construct in the presence of lithium chloride and increasing concentrations of IS, IA, QA, AA, XA, Kyn and KA. RLU was measured. Student's t-test with Bonferroni correction was applied for multiple comparisons. Compared to lithium chloride alone, p values were < 0.0125 for individual concentrations tested for IS, IA, Kyn and KA. For simplicity, the p values are shown bracketed together in graphs.

Supplemental figure 8.

(A). ECs were treated with pooled human control sera at the final concentration of 5% spiked with the indicated concentration of Kyn for 16 hours, after which the cells underwent subcellular fractionation. The fractions were probed for β-catenin. Tubulin and fibrillarin were probed as the

loading control and the markers of cytosol and nuclear fractions, respectively. Representative images of three independent experiments are shown.

(B). β-catenin was quantitated using ImageJ and normalized to loading controls. Average of normalized β-catenin from three experiments is shown. The cytosol and nuclear fractions were analyzed separately and compared to the control (Kyn = 0 μM) using Student's t-test with Bonferroni's correction of multiple comparisons._Error bars = SD. Compared to vehicle-treated cells, *** $P < 0.001$ for Kyn at all concentrations for their both the fractions.

(C). ECs were treated with vehicle or Kyn at the indicated concentration and exposed to MG132 10 μM overnight before harvest. Immunoprecipitation of the lysates was performed using anti- β-catenin antibody and eluents were probed with anti-ubiquitin antibody. The blot was stripped and reprobed with anti- β-catenin antibody. Representative images of three independent experiments are shown.

(D). The ubiquitinated β-catenin was quantitated using ImageJ and normalized to the immunoprecipitated β-catenin. Average of normalized ubiquitinated β-catenin from three experiments is shown. Student's t-test with Bonferroni correction was performed._Error bars = SD. Compared to vehicle-treated cells, **$p = 0.004$ and *** $p < 0.001$. The dotted blue lines correspond to the concentration of Kyn at different stages of CKD. Please see Table S2 for further detail.

Supplemental figure 9

(A). ECs transduced with lentiviral particles were selected with puromycin. The cell lysates were probed for AHR and GAPDH served as a loading control. Representative images of three independent experiments are shown.

(B). Densitometry analysis was performed on ECs transduced with control or AHR CRISPR lentiviral particles. The lysates probed for AHR. AHR normalized with GAPDH was performed.

Average of three independent experiments are shown. Scale bar= SEM. Student's t-test was performed. *** $p < 0.0001$.

(C). The lysates from ECs knocked out of *AHR* (AHR CRISPR) were probed for β-catenin. Actin served as a loading control. Representative images of three independent experiments are shown.

(D). Densitometry analysis of β-catenin normalized with Actin was performed. Average of three independent experiments are shown. Scale bar = SEM. Student's t-test was performed. *** $p < 0.0001$.

(E). The ECs knocked out of *AHR* or control cells were treated with 5% pooled uremic serum or control sera. The lysates were probed for β-catenin. Actin served as a loading control. Representative images of three independent experiments are shown.

(F). Densitometry analysis of β-catenin normalized with Actin in cells treated with human sera was performed. Average of three independent experiments are shown. Scale bar= SEM. Student's t-test was performed. * $p = 0.048$, ## $p = 0.006$. *** $p = 0.003$.

Supplemental figure 10

(A). Fli-eGFP transgenic zebrafish were treated with IS at the indicated concentration at 24 hours post fertilization (hpf) for additional 48 hours. DMSO-treated embryos served as controls. The water was replenished with fresh water daily. Representative images taken under the same microscope settings were obtained from randomly selected fish from a total of twenty embryos in each group. The blue arrowheads mark the branching of intersegmental vessels. Scale bar = 10 microns.

(B). Fli-eGFP transgenic zebrafish were treated with IS as above. Representative images taken under the same microscope settings were obtained from randomly selected fish from a total of

twenty embryos in each group. The white arrowheads mark the branching of intersegmental vessels and the blue arrowhead point to thin intersegmental vessels in IS-treated fish. Scale bar = 10 microns.

(C). Fli-eGFP transgenic zebrafish were treated with IS at 24 hpf and for 48 additional hours as above. Representative images taken under the same microscope settings were obtained from randomly selected fish from a total of ten embryos in each group. Scale bar = 10 microns.

(D). Images of the Fli-eGFP transgenic zebrafish treated with indicated concentrations of IS were analyzed using ImageJ. The eGFP signal was quantified using integrated density in the marked region of interest and normalized to the surface area in micron^2 and presented as a box plot. A Student's t-test with Bonferroni correction was performed to compare the groups. Compared to the DMSO-treated zebrafish embryos, $p = 0.012$ and $p = 0.002$ for IS at 50 µM and 100 µM concentrations, respectively.

Supplemental figure 11

(**A**). Blood urea nitrogen (BUN) levels in the two groups of mice at the end of the experiment are shown. Student's t-test was performed. N =7 mice per group.

(B). IS levels were measured using pre-validated LC/MS in blood of control and IS-administered mice at the end of the experiments and shown as a scatter plot. The line in each group represent median. Student's t test was performed to compare both the groups. ***$p < 0.001$. N =7 mice per group.

Supplemental figure 12

(A). Representative images of hematoxylin and eosin stained kidneys from ten mice from both the groups are shown. Scale bar = 25 microns.

(B). Kidney injury, estimated by interstitial fibrosis and tubular atrophy (IFTA), was compared (see methods for details) between both groups. Student's t-test was applied. ***p < 0.001. N =10 mice per group.

(**C**) Blood urea nitrogen (BUN) levels in the two groups of mice at the end of the experiment are shown as box plots. N =10 mice per group. Student's t-test was performed. ** p = 0.005.

(D). Creatinine levels in the two groups of mice at the end of the experiment are shown as box plots. The line within the box corresponds to the median, and the box represents the 25th and 75th percentiles and whisker results in minimum and maximum values. N =10 mice per group. Student's t-test was performed. *** p < 0.001.

(**E**). IS levels were measured using pre-validated LC/MS in blood of mice from normal diet and adenine diet at the end of the experiments and shown as a scatter plot. The line in each group represent median. Student's t test was performed to compare both the groups. ***p <0.001. N =10 mice per group.

(F). The posterior calf muscles of the ligated limb of mice on normal diet and adenine diet were probed for β-catenin. Ponceau served as loading control. Representative immunoblots from three separate mice from each group of (N = 10 mice/group) are shown. N =10 mice per group

(**G**). Densitometry analysis was performed on β-catenin band from the muscle lysates of mice from both the groups and represented as a scatter plot. The line in each group represent median. Student's t test was performed to compare both the groups. **p <0.01. N =10 mice per group

(**H-I**). ECs stably expressing XRE-responsive promoter tethered to luciferase reporter (**G**) and LS construct were treated with sera from mice from adenine group (N= 10) **(H).** Mice exposed to normal diet served as controls (N= 10). The luciferase assay was performed and normalized to the protein content. Line corresponds to median value. Groups were compared using Student's t-test.

(J). A correlation analyses between the AHR and Wnt activity in ECs in response to sera from CKD mice. **p <0.01, ***p <0.001. N = 10 mice per group.

Supplemental figure 13

(**A**). Kyn levels were measured using pre-validated LC/MS in blood of mice from normal diet and adenine diet at the end of the experiments and shown as a scatter plot. The line in each group represent median. Student's t test was performed to compare both the groups. **p 0.013. N =10 mice per group.

(B-D). Correlation analyses performed between the levels of Kyn in the adenine mice with normalized CD31 ($R^2 = 0.296$, $p = 0.025$) (**B**), normalized β-catenin expression in the capillaries of muscles ($R^2 = 0.353$, $p = 0.041$) (**C**), with VEGF-A expression in the capillaries ($R^2 = 0.387$, $p < 0.001$) (**D**).

(**E**). A correlation analysis was performed. Wnt activity in EC in response to serum from individual CKD mice were correlated with their Kyn levels in blood. Their linear regression analyses are shown. $R^2 = 0.444$, $p = 0.035$.

Supplemental figure 14

(A) BUN values of mice from four groups shown in Fig. 7 at the end of experiment are shown. The line in each group corresponds to the median. Independent Student's t-tests were applied. No significant differences were observed between control v/s control + CH223191 group, and adenine v/s adenine + CH223191 group. N= 8 mice/group.

(B) Average perfusion ratios (ligated/non-ligated limb) from eight mice on normal diet (non-CKD)/group is shown. Error bars = SD. Independent Student's t-tests were applied to compare

groups at each time point. Compared to mice on vehicle diet, mice with CH223191 *p = 0.012 in day 21.

(**C-E**). Integrated density analysis was conducted for CD31 and α-Actin. The integrated density of CD31+ was normalized to the corresponding α-Actin images and presented as a ratio **(C),** normalized β-catenin in capillary and muscles **(D)** and VEGF **(E).** Eight mice per group are shown. Student's t-test was performed. * p = 0.041 (CD31/SMA+) *p = 0.026 β-catenin in capillary and p = 0.006 for β-catenin in muscles. Student's t-test was applied. * p = 0.053 VEGFA/CD31+.

Supplemental figure 15

(A-B). Correlation analyses were performed. Wnt activity in EC in response to serum from individual CKD patients were correlated with their respective IS levels (A) and Kyn levels (B). Their linear regression analyses are shown. IS $R^2 = 0.34$, $p < 0.001$ and Kyn $R^2 = 0.25$, $p = 0.025$.

References:

1. Shashar M, Belghasem ME, Matsuura S, Walker J, Richards S, Alousi F, et al. Targeting STUB1-tissue factor axis normalizes hyperthrombotic uremic phenotype without increasing bleeding risk. *Sci Transl Med.* 2017;9(417):1-11.
2. Belghasem ME, A'Amar O, Roth D, Walker J, Arinze N, Richards SM, et al. Towards minimally-invasive, quantitative assessment of chronic kidney disease using optical spectroscopy. *Sci Rep.* 2019;9(1):7168.
3. Zhang A RK, , Ng Seng Kah, Ravid K and Chitalia V. A Mass Spectrometric Method for Quantification of Tryptophan-Derived Uremic Solutes in Human Serum *Journal of Biological Methods.* 2017;epub:1-8.
4. Kolachalama VB, Shashar M, Alousi F, Shivanna S, Rijal K, Belghasem ME, et al. Uremic Solute-Aryl Hydrocarbon Receptor-Tissue Factor Axis Associates with Thrombosis after Vascular Injury in Humans. *J Am Soc Nephrol.* 2018;29(3):1063-72.
5. Schneider CA, Rasband WS, and Eliceiri KW. NIH Image to ImageJ: 25 years of image analysis. *Nat Methods.* 2012;9(7):671-5.
6. Watanabe Y, Murdoch CE, Sano S, Ido Y, Bachschmid MM, Cohen RA, et al. Glutathione adducts induced by ischemia and deletion of glutaredoxin-1 stabilize HIF-1alpha and improve limb revascularization. *Proc Natl Acad Sci U S A.* 2016;113(21):6011-6.
7. Mamchaoui K, Trollet C, Bigot A, Negroni E, Chaouch S, Wolff A, et al. Immortalized pathological human myoblasts: towards a universal tool for the study of neuromuscular disorders. *Skelet Muscle.* 2011;1:34.
8. Sanjana NE, Shalem O, and Zhang F. Improved vectors and genome-wide libraries for CRISPR screening. *Nat Methods.* 2014;11(8):783-4.
9. Chitalia VC, Shivanna S, Martorell J, Balcells M, Bosch I, Kolandaivelu K, et al. Uremic serum and solutes increase post-vascular interventional thrombotic risk through altered stability of smooth muscle cell tissue factor. *Circulation.* 2013;127(3):365-76.
10. Chitalia VC, Foy RL, Bachschmid MM, Zeng L, Panchenko MV, Zhou MI, et al. Jade-1 inhibits Wnt signalling by ubiquitylating beta-catenin and mediates Wnt pathway inhibition by pVHL. *Nat Cell Biol.* 2008;10(10):1208-16.
11. Chitalia V, Shivanna S, Martorell J, Meyer R, Edelman E, and Rahimi N. c-Cbl, a ubiquitin E3 ligase that targets active beta-catenin: a novel layer of Wnt signaling regulation. *J Biol Chem.* 2013;288(32):23505-17.
12. Shashar M, Siwak J, Tapan U, Lee SY, Meyer RD, Parrack P, et al. c-Cbl mediates the degradation of tumorigenic nuclear beta-catenin contributing to the heterogeneity in Wnt activity in colorectal tumors. *Oncotarget.* 2016;7(44):71136-50.
13. Shivanna S, Harrold I, Shashar M, Meyer R, Kiang C, Francis J, et al. The c-Cbl ubiquitin ligase regulates nuclear beta-catenin and angiogenesis by its tyrosine phosphorylation mediated through the Wnt signaling pathway. *J Biol Chem.* 2015;290(20):12537-46.
14. Shivanna S, Kolandaivelu K, Shashar M, Belghasim M, Al-Rabadi L, Balcells M, et al. The Aryl Hydrocarbon Receptor is a Critical Regulator of Tissue Factor Stability and an Antithrombotic Target in Uremia. *J Am Soc Nephrol.* 2016;27(1):189-201.

15. Parks AJ, Pollastri MP, Hahn ME, Stanford EA, Novikov O, Franks DG, et al. In silico identification of an aryl hydrocarbon receptor antagonist with biological activity in vitro and in vivo. *Mol Pharmacol.* 2014;86(5):593-608.
16. Vanholder R, Schepers E, Pletinck A, Nagler EV, and Glorieux G. The uremic toxicity of indoxyl sulfate and p-cresyl sulfate: a systematic review. *J Am Soc Nephrol.* 2014;25(9):1897-907.
17. Hartsough EJ, Meyer RD, Chitalia V, Jiang Y, Marquez VE, Zhdanova IV, et al. Lysine methylation promotes VEGFR-2 activation and angiogenesis. *Sci Signal.* 2013;6(304):ra104.
18. Barreto FC, Barreto DV, Liabeuf S, Meert N, Glorieux G, Temmar M, et al. Serum indoxyl sulfate is associated with vascular disease and mortality in chronic kidney disease patients. *Clin J Am Soc Nephrol.* 2009;4(10):1551-8.
19. Debnath S, Velagapudi C, Redus L, Thameem F, Kasinath B, Hura CE, et al. Tryptophan Metabolism in Patients With Chronic Kidney Disease Secondary to Type 2 Diabetes: Relationship to Inflammatory Markers. *Int J Tryptophan Res.* 2017;10:1178646917694600.

Supplemental figure 1

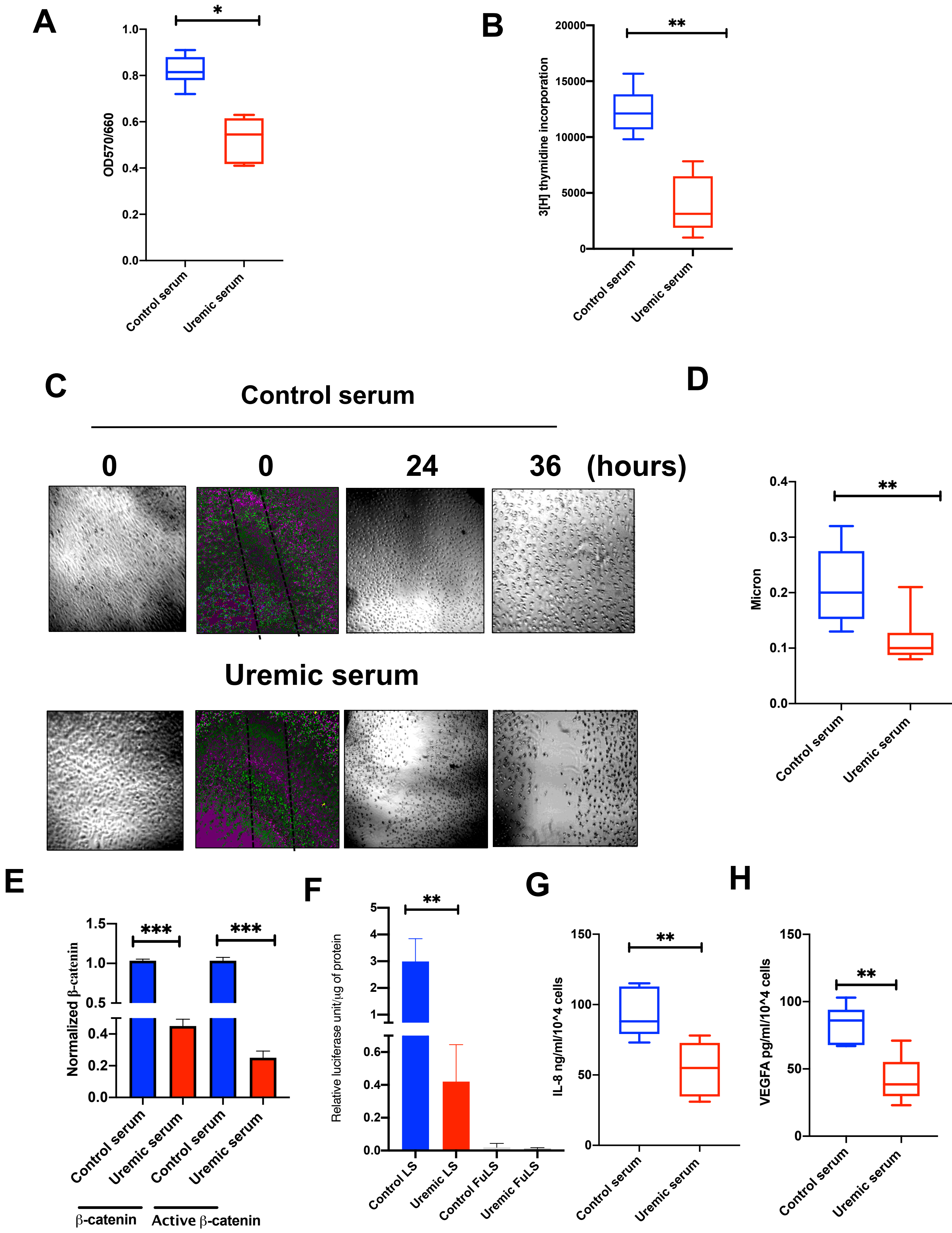

Supplemental Figure 1

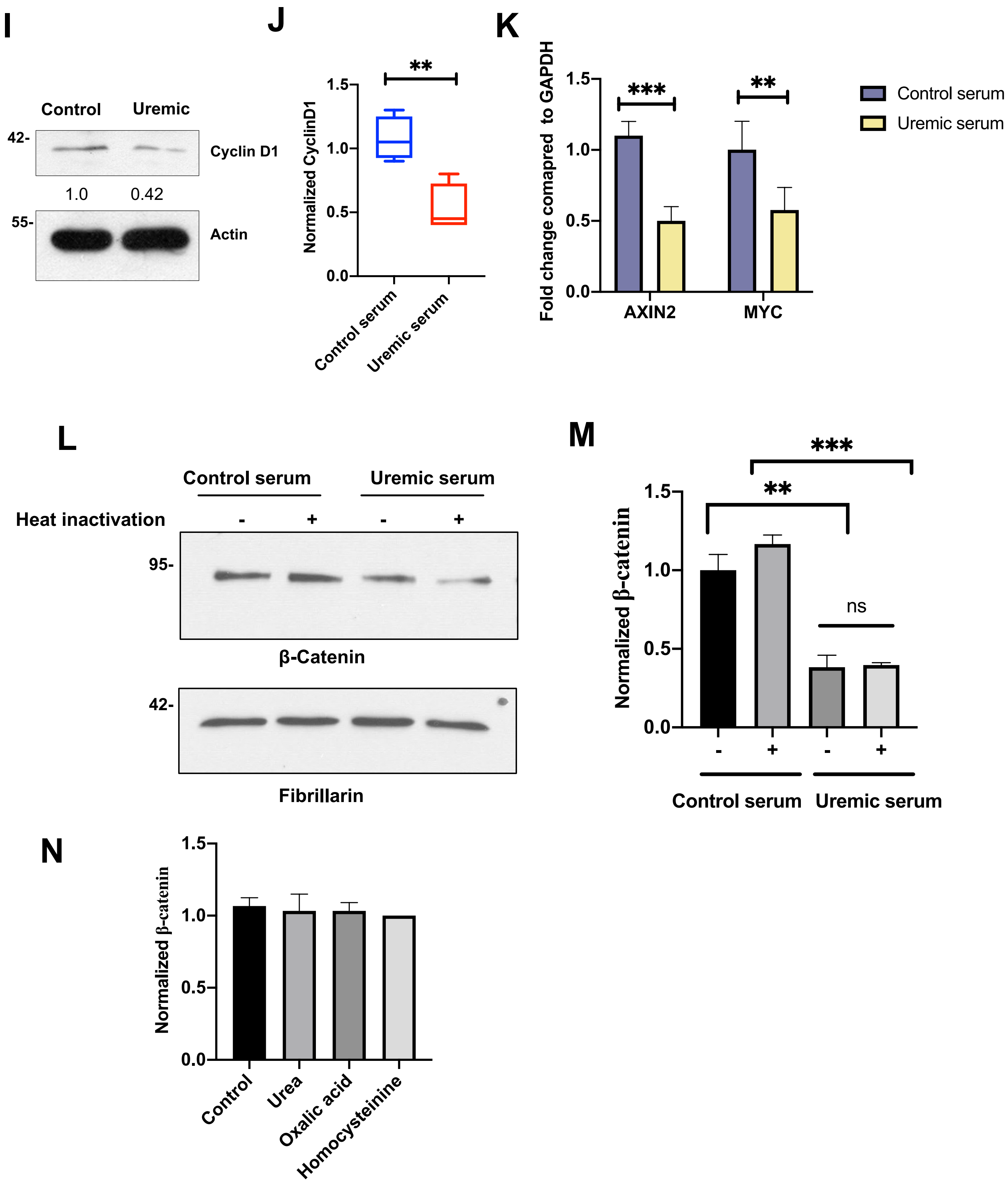

Supplemental Figure 2

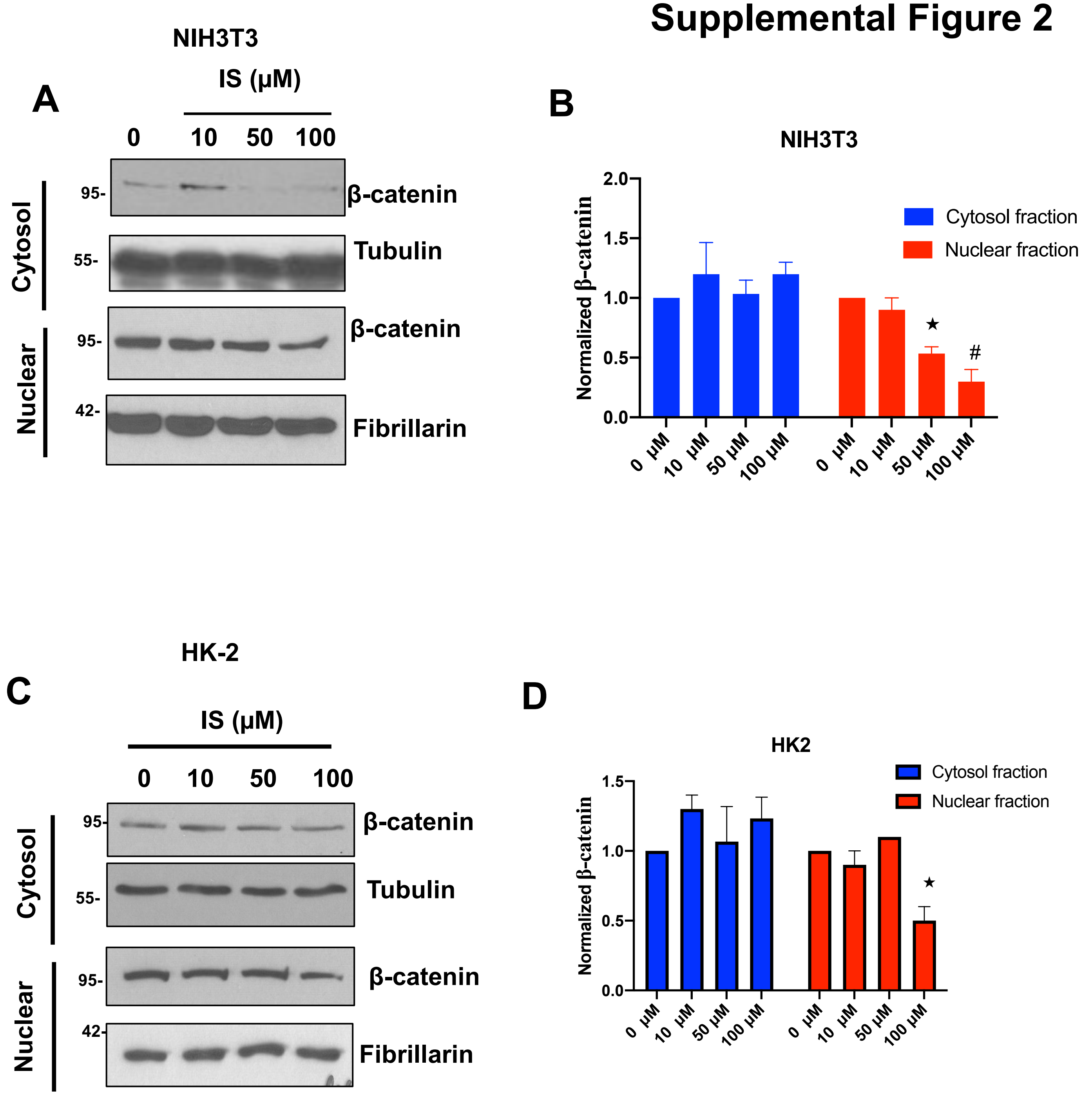

Supplemental Figure 2

E

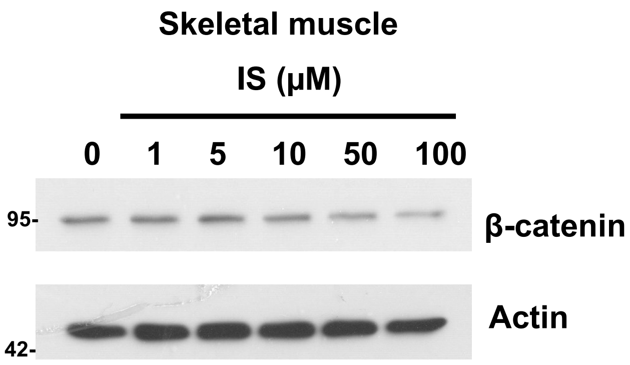

F

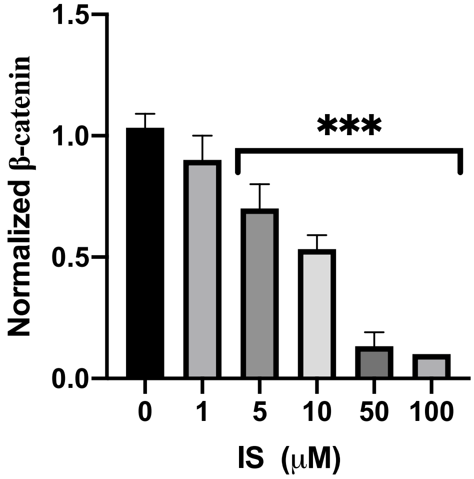

Supplemental Figure 3

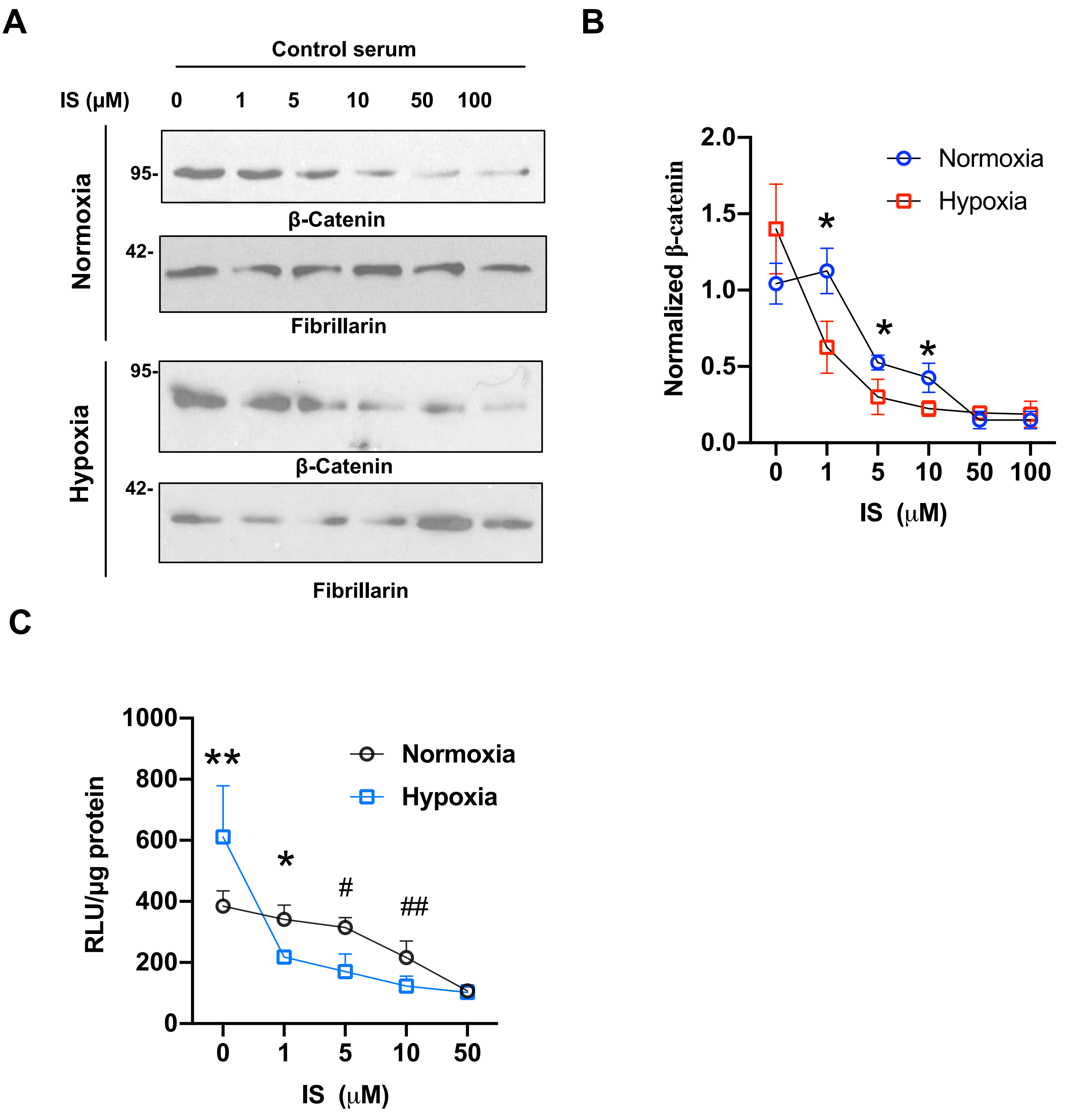

Supplemental Figure 4

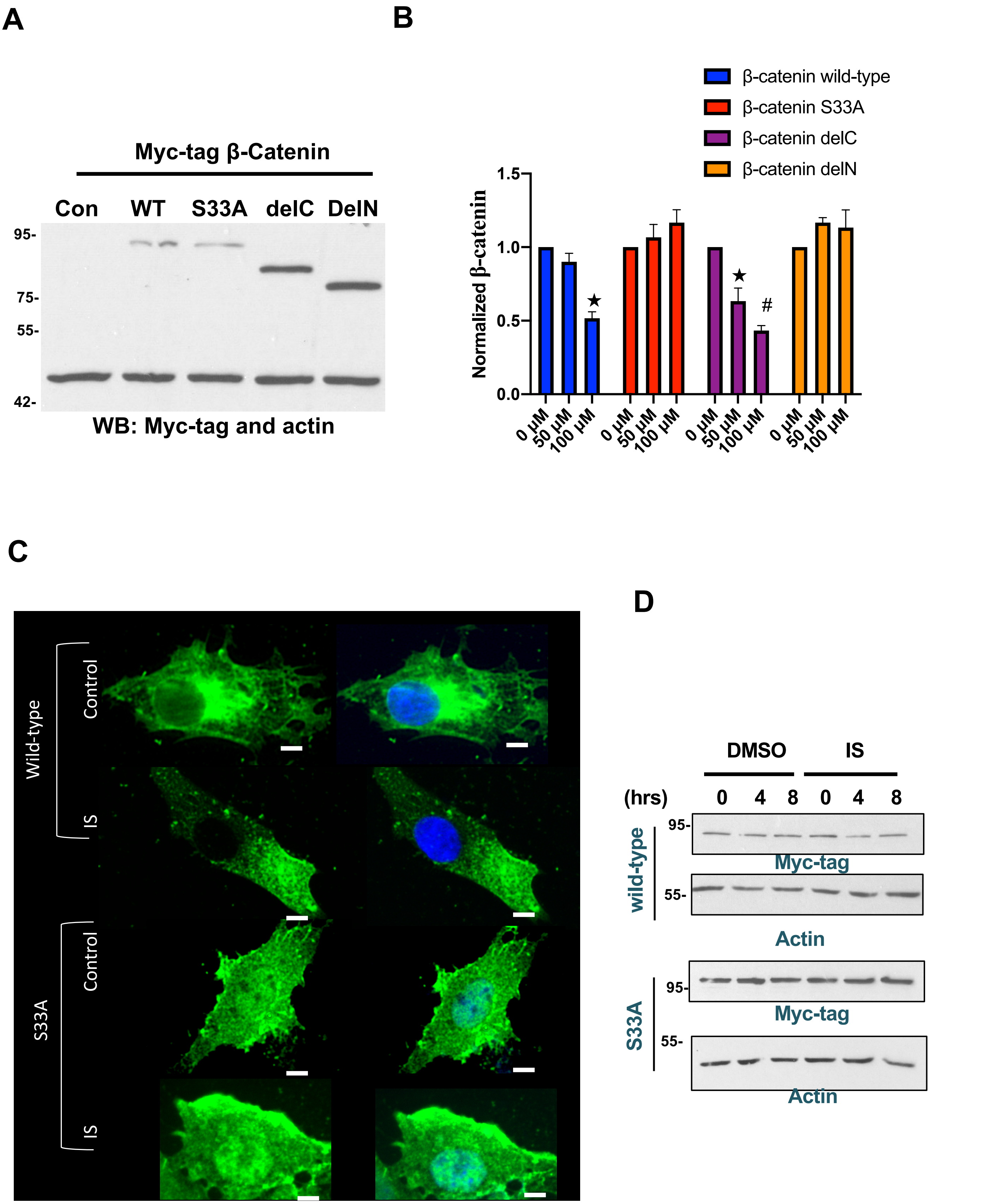

Supplemental Figure 5

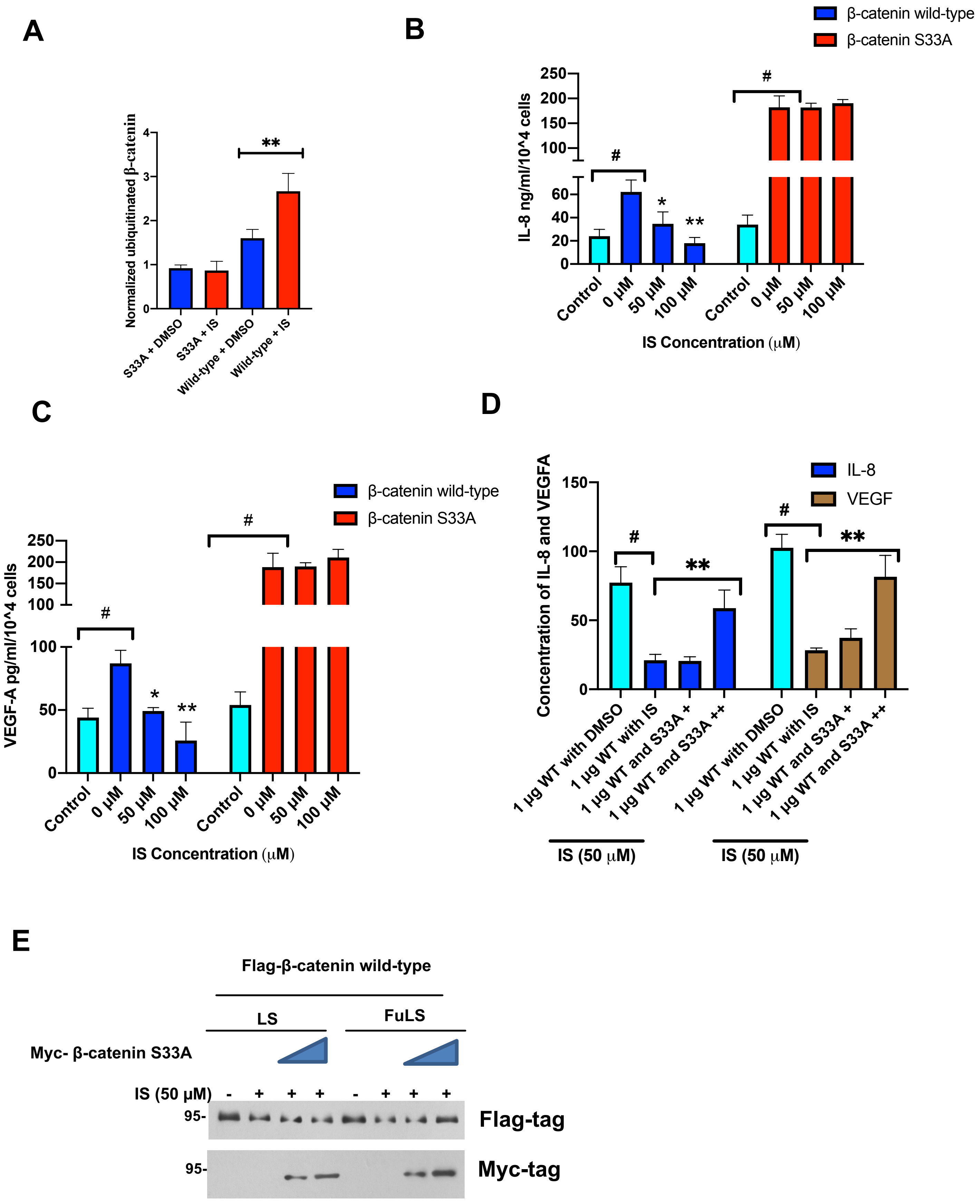

Supplemental Figure 6

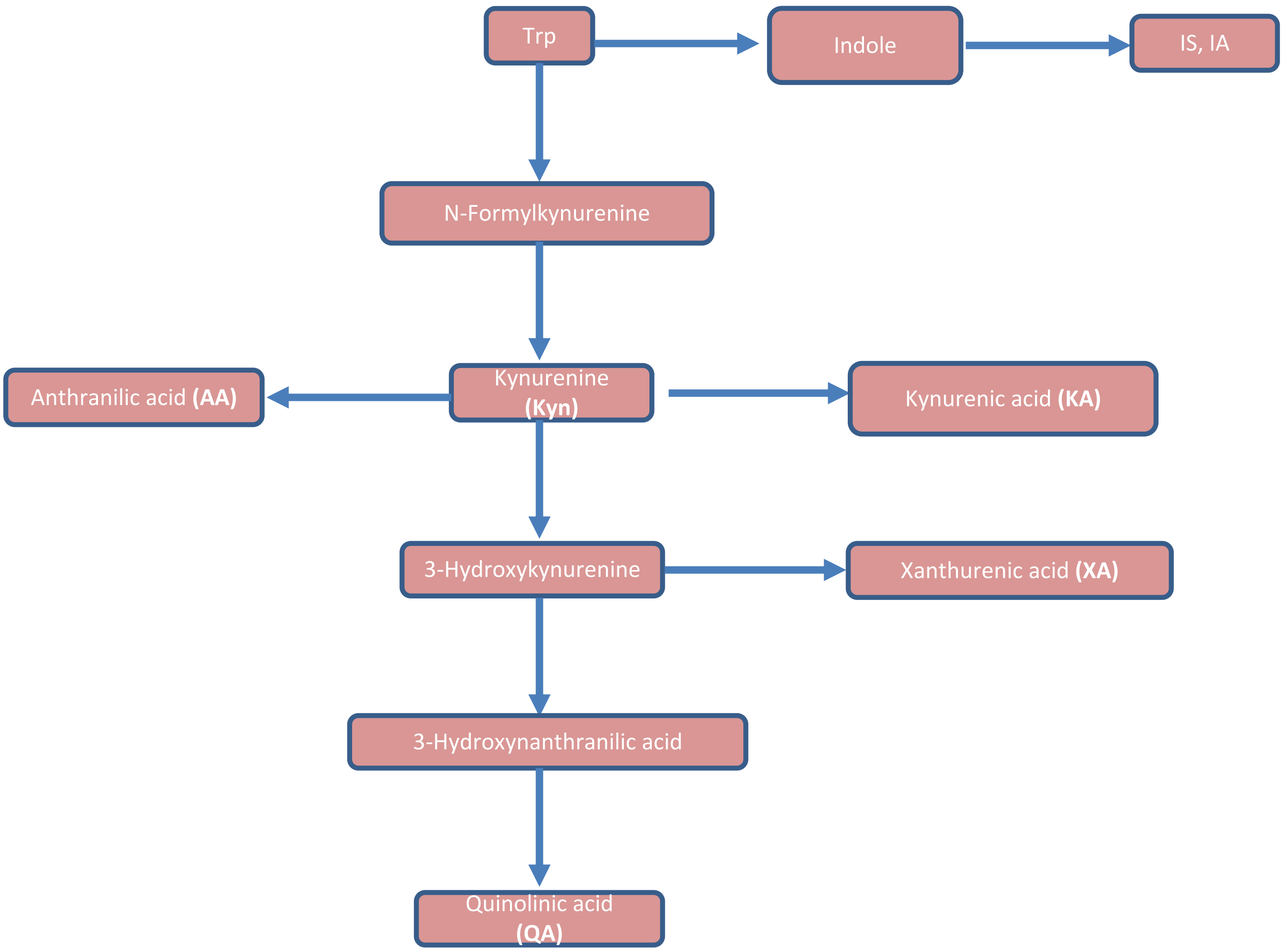

Supplemental Figure 7

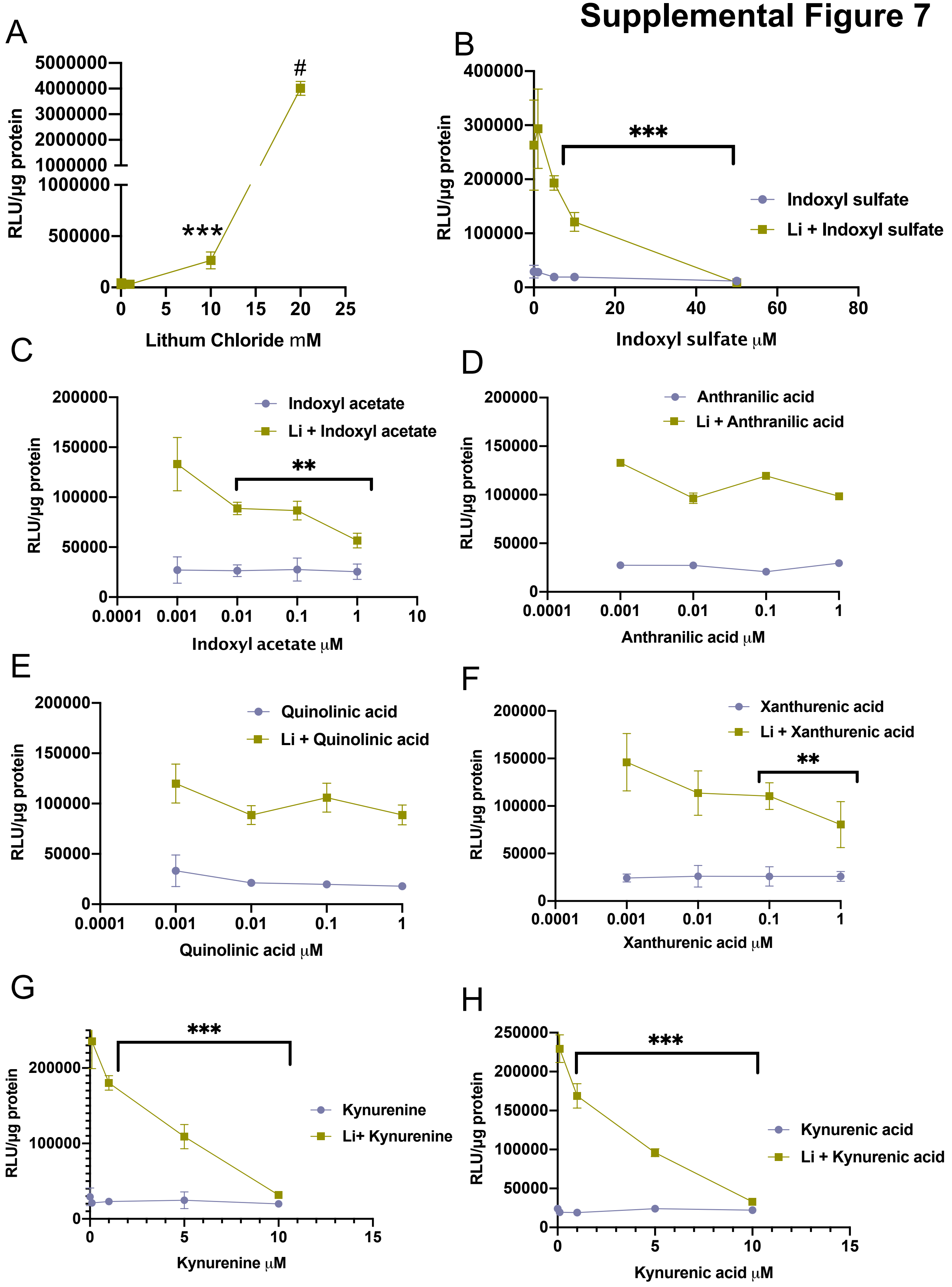

Supplemental Figure 8

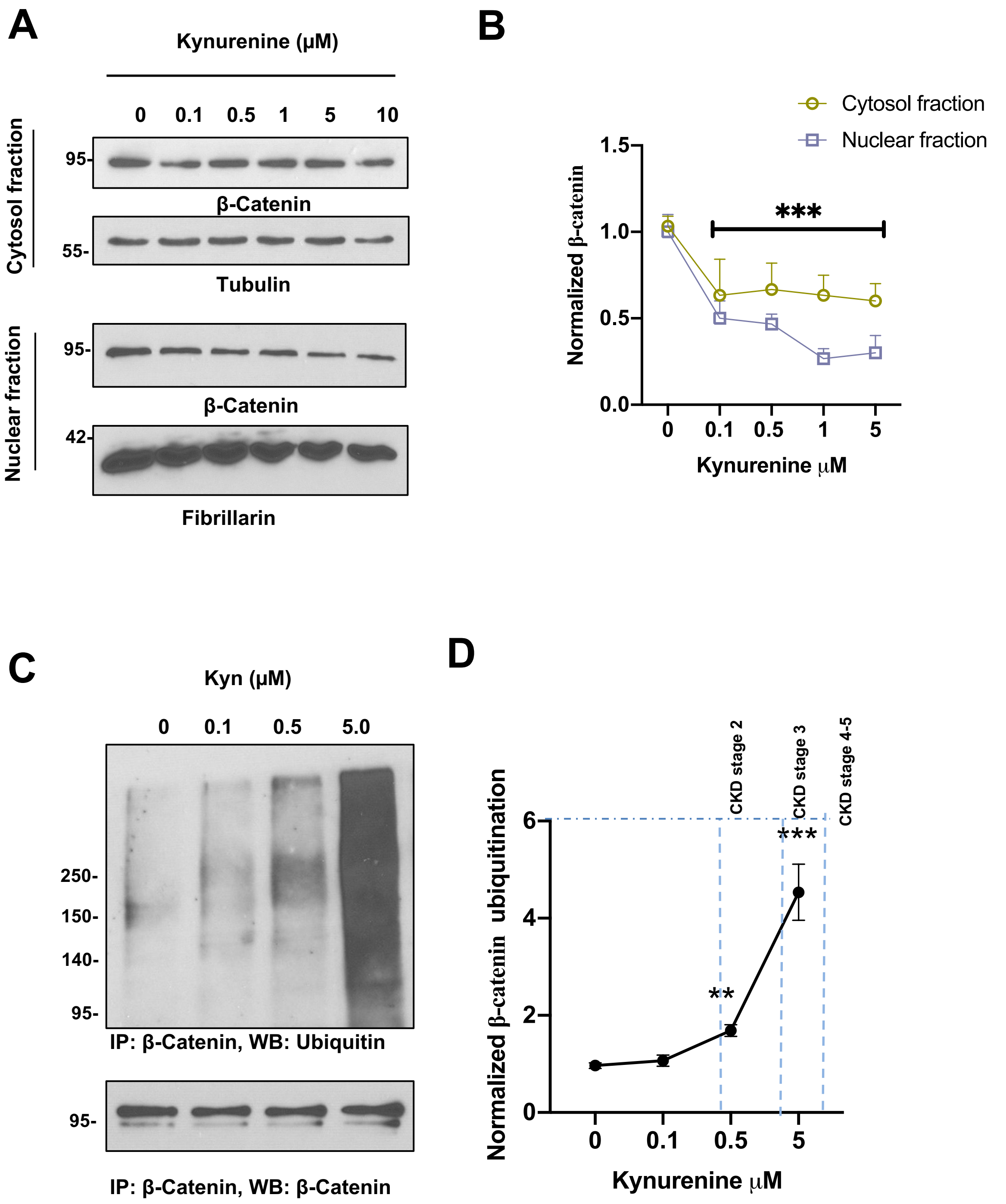

Supplemental Figure 9

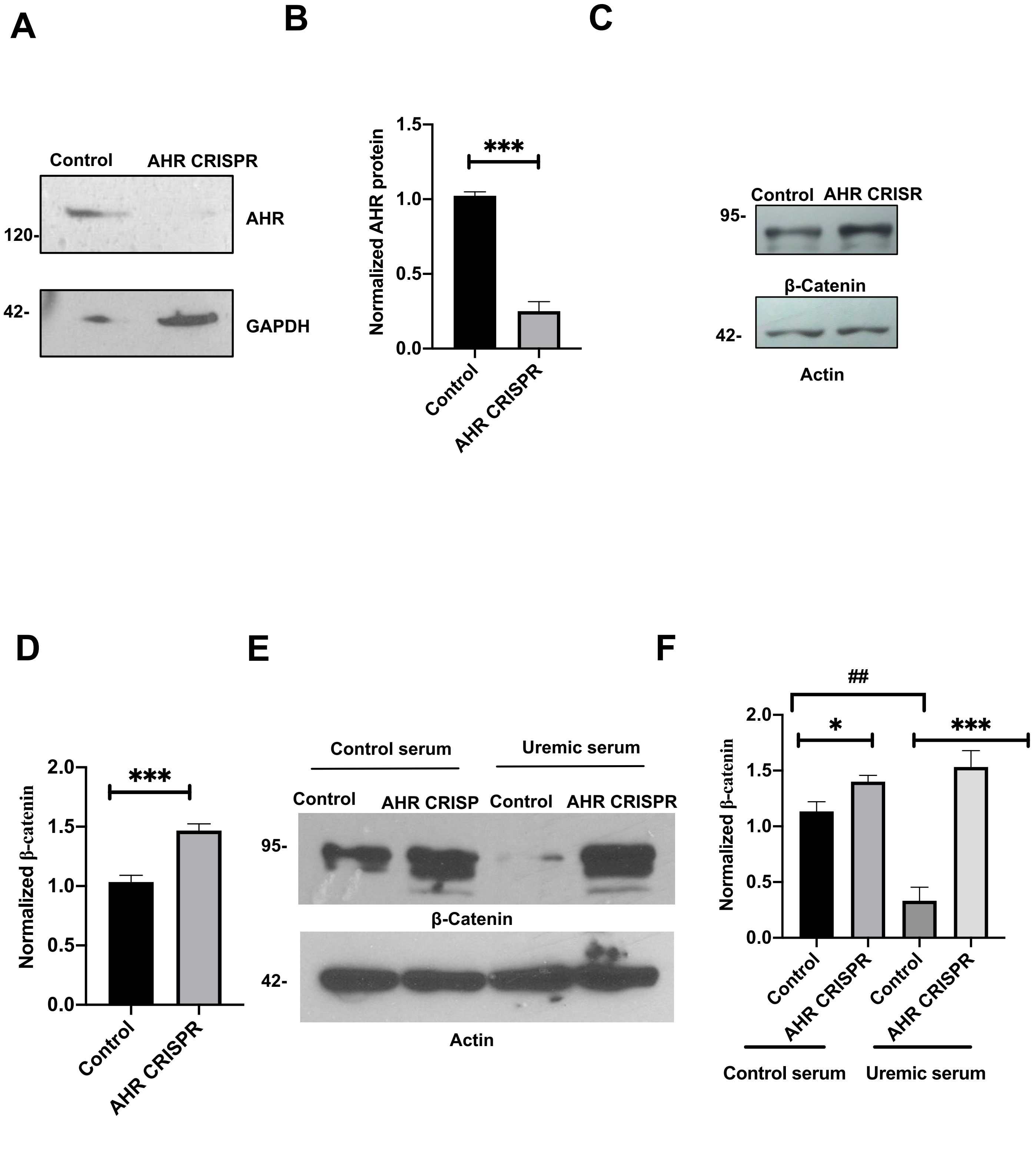

Supplemental Figure 10

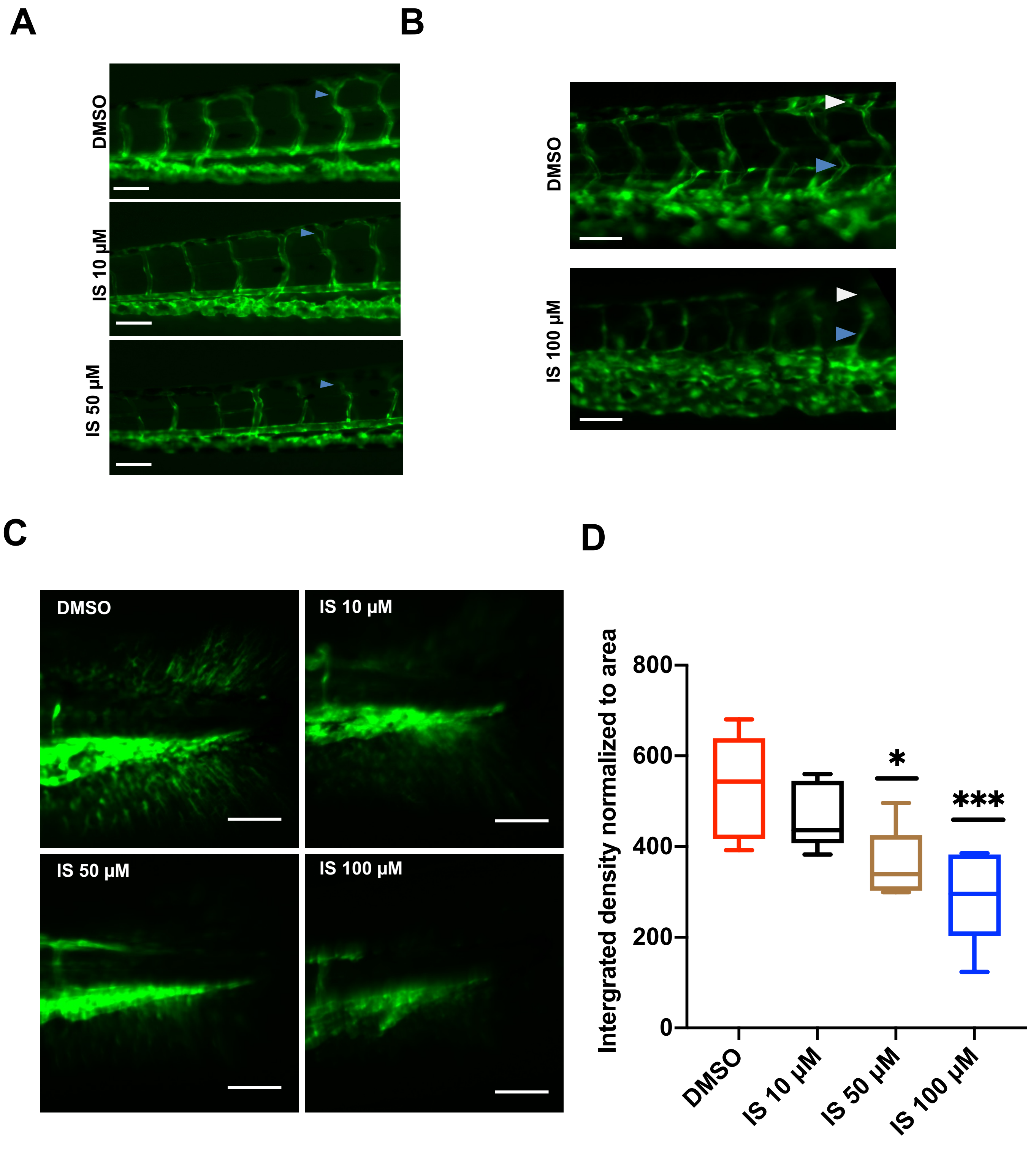

Supplemental Figure 11

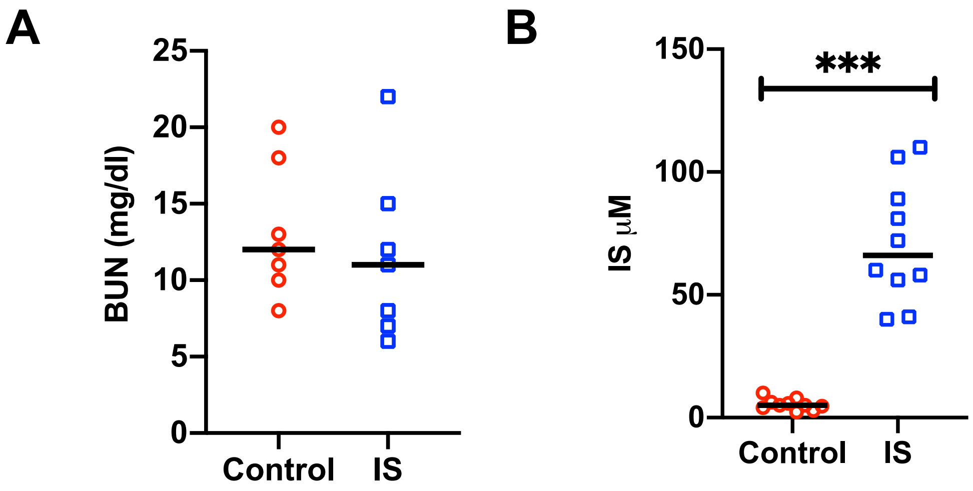

Supplemental Figure 12

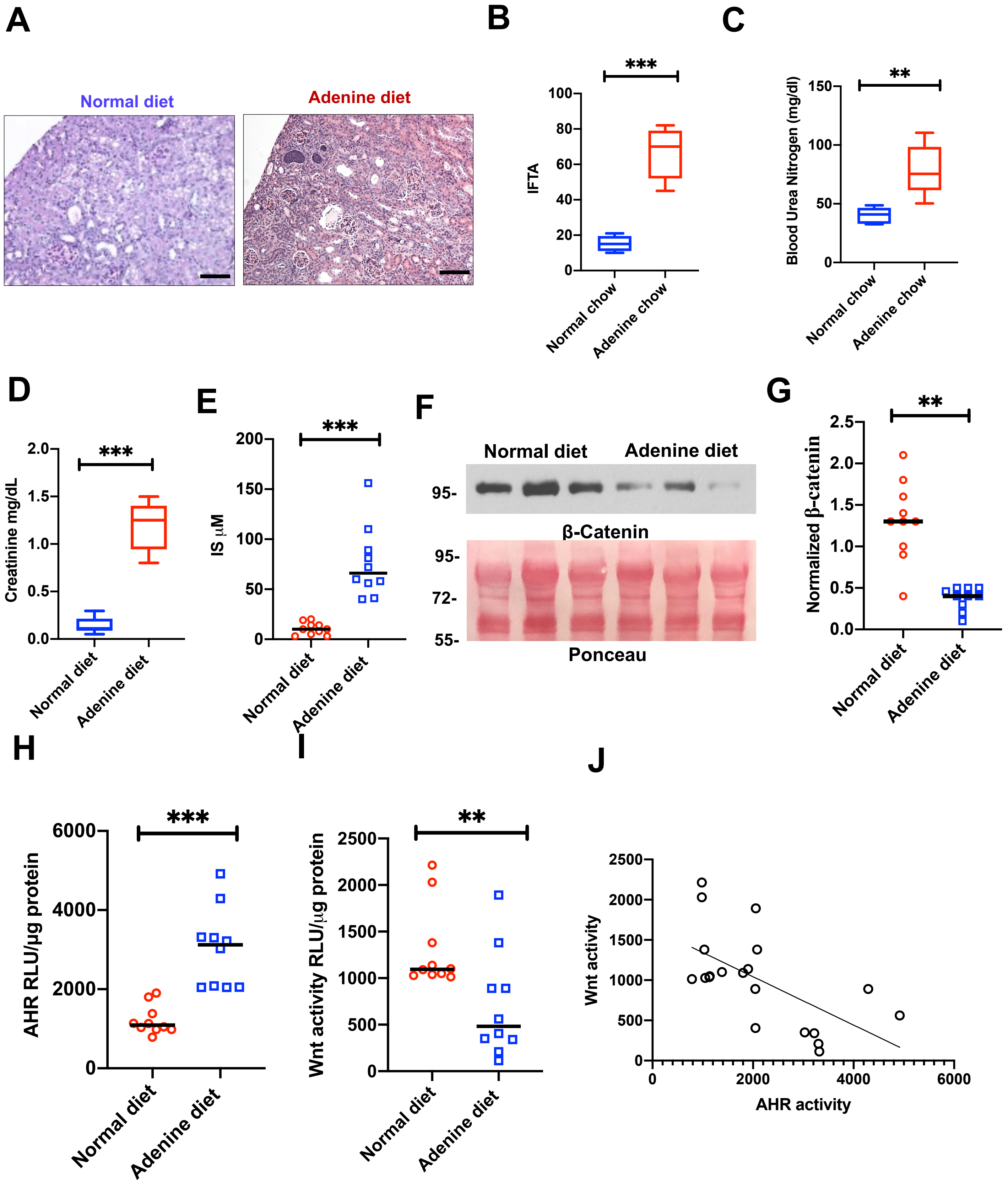

Supplemental Figure 13

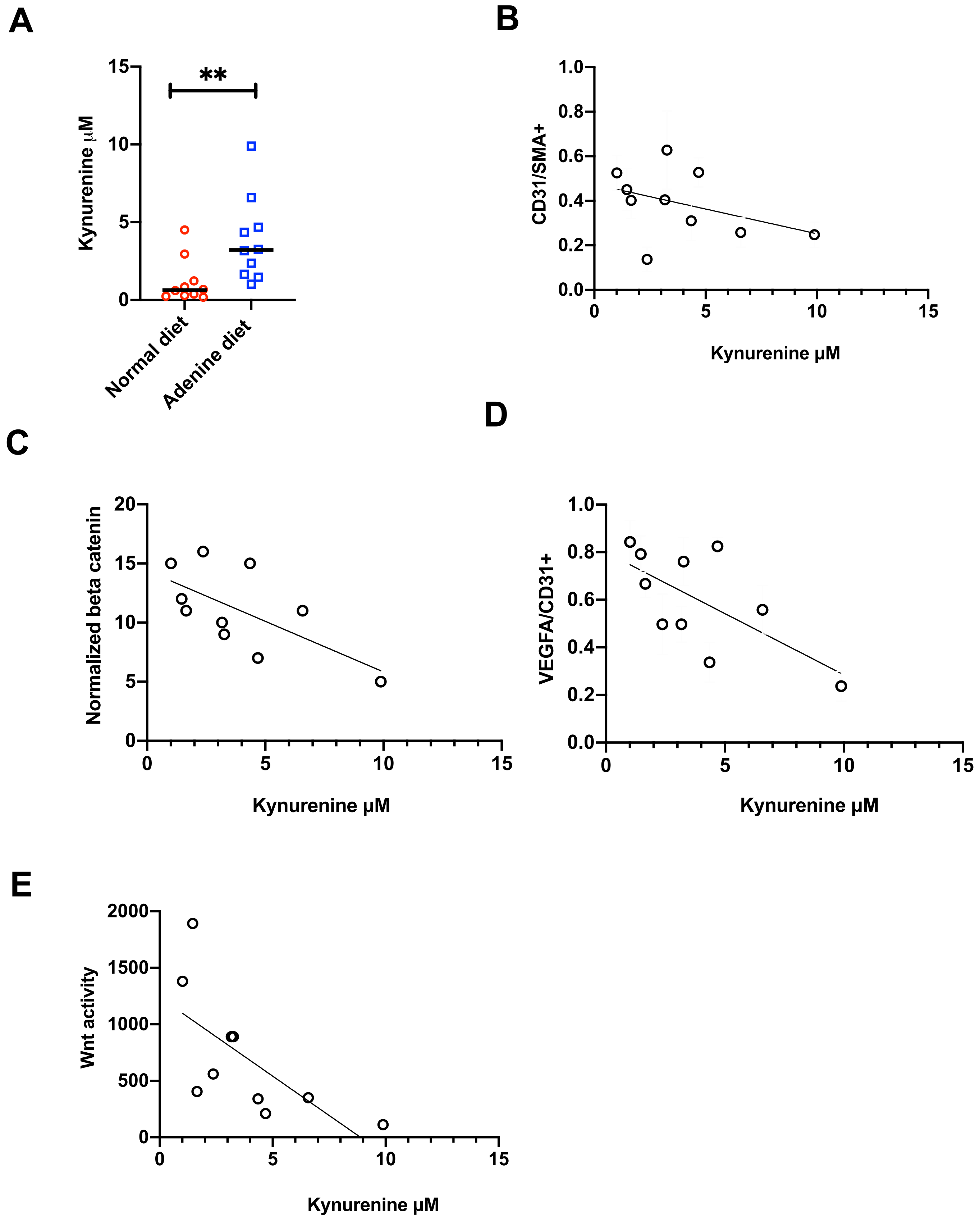

Supplemental Figure 14

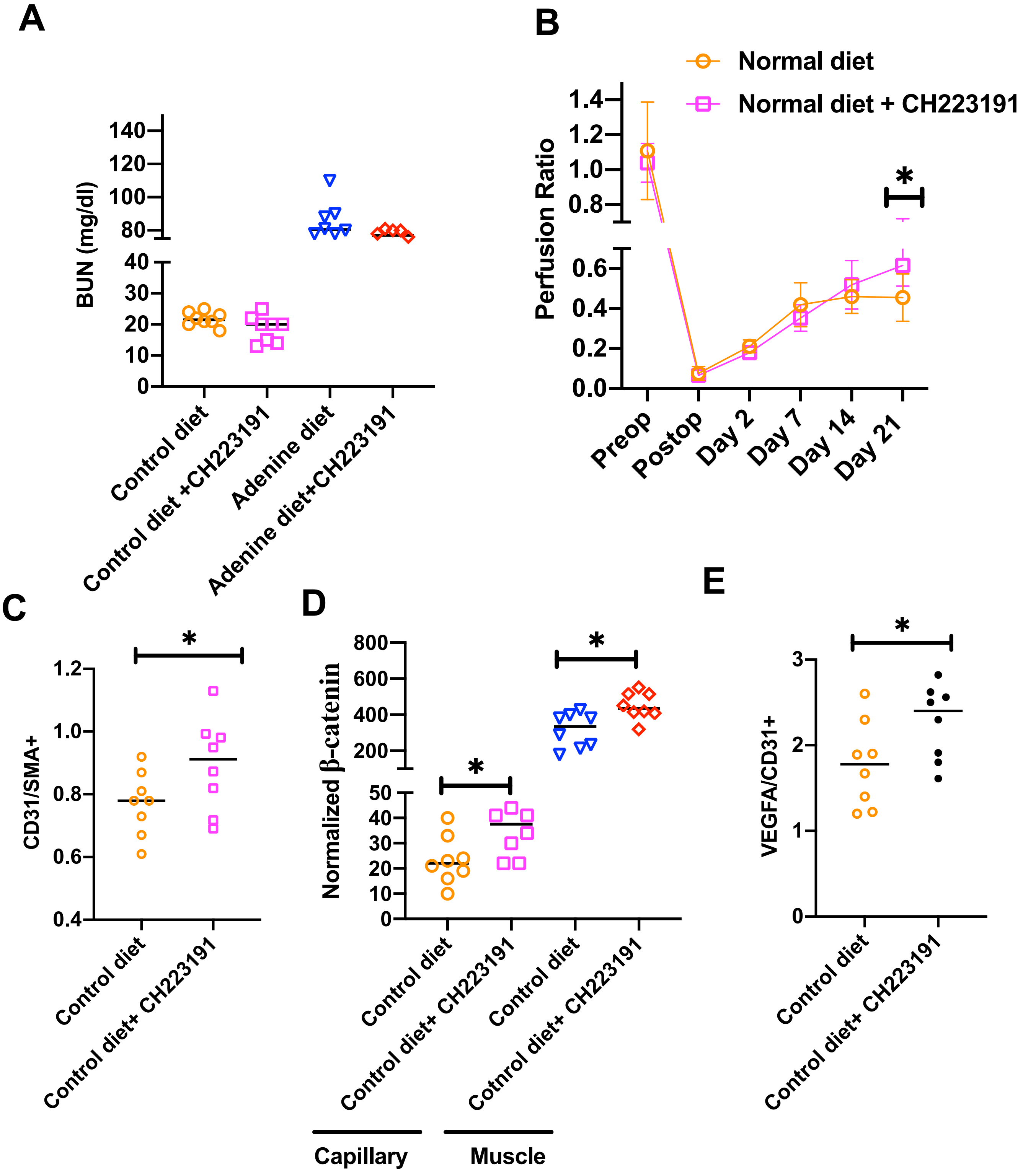

Supplemental Figure 15

A

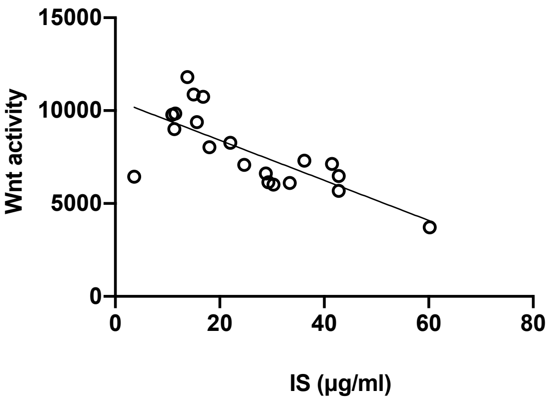

B

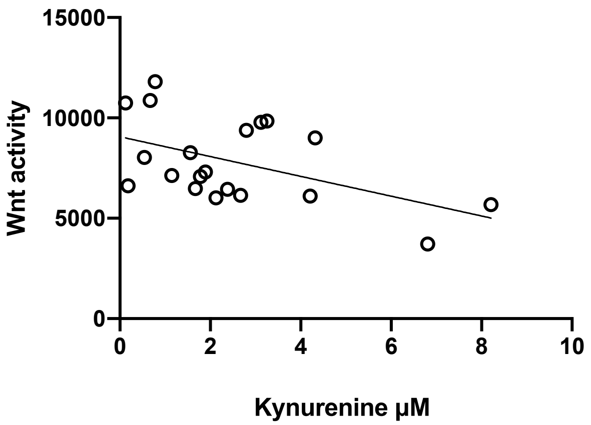

Supplement: Supplemental data [file jci-132-142260-s103.pdf]
